# Quality of life in individuals at clinical high risk for psychosis: A systematic review and meta-analysis

**DOI:** 10.1192/j.eurpsy.2026.10170

**Published:** 2026-02-18

**Authors:** Jesús Camacho, Javier de Otazu Olivares, Omar Ríos Hernández, Claudia Aymerich, Grace Frearson, Ana Catalan, Gonzalo Salazar de Pablo

**Affiliations:** 1 Institute of Psychiatry and Mental Health, Hospital Clínico San Carlos Universidad Complutense, Spain; 2 Department of Child and Adolescent Psychiatry, Institute of Psychiatry, Psychology & Neuroscience King’s College London, UK; 3 School of Medicine, Universidad Nacional Pedro Henriquez Urena, Dominican Republic; 4 Psychology Department, Universidad of Valencia, Spain; 5 Consorcio Hospitalario Provincial de Castellón, Castellón, Spain; 6 Psychiatry Department, Biocruces Bizkaia Health Research Institute, OSI Bilbao- Basurto. Facultad de Me, Barakaldo, Spain; 7 CIBERSAM (Centro de Investigación en Red de Salud Mental), Instituto de Salud Carlos III, Madrid, Spain; 8 Department of Psychosis Studies, Institute of Psychiatry, Psychology and Neuroscience King’s College London, UK; 9 Child and Adolescent Mental Health Services, South London and Maudsley NHS Foundation Trust, London, UK; 10 Department of Child and Adolescent Psychiatry, Institute of Psychiatry and Mental Health, Hospital General Universitario "G. Marañón" School of Medicine, Universidad Complutense, Madrid, Spain

**Keywords:** clinical high risk, evidence, meta-analysis, quality of life, systematic review

## Abstract

**Background:**

Quality of life (QoL) is an important clinical outcome in mental health. However, evidence on its progression and predictors in individuals at clinical high risk for psychosis (CHR-P) remains limited. This meta-analysis examined correlates, impact, and longitudinal changes in QoL among CHR-P individuals.

**Methods:**

Following PRISMA guidelines (PROSPERO: CRD42024560092), we systematically searched multiple databases from inception to 27 May 2025. Eligible studies included CHR-P participants assessed with validated QoL instruments. Data extraction was performed independently, and meta-analyses, meta-regressions, heterogeneity, and publication bias analyses were conducted. Risk of bias was evaluated using a modified Newcastle–Ottawa Scale (NOS).

**Results:**

Thirty-one studies were included (2,288 CHR-P individuals; mean age = 21.47 years; 48.9% female). Compared with healthy controls (HC), CHR-P participants showed significantly poorer QoL (Hedges’ g = 1.39, 95% CI = 0.97–1.83). Longitudinal analyses indicated QoL improvements after 1 year (Hedges’ g = 1.40, 95% CI = 0.74–2.07) and at 2–3 years follow-up (Hedges’ g = 3.24, 95% CI = 0.73–5.75). Substantial heterogeneity was observed across analyses. Meta-regressions showed no significant influence of age, sex, functioning, symptom severity, or study quality. Study quality scores ranged from 1 to 8 (median = 6, mean = 5.8, SD = 1.13).

**Conclusions:**

Individuals at CHR-P present markedly poorer QoL compared with healthy controls. Longitudinal data suggest possible improvements over time; however, heterogeneity and limited long-term evidence warrant cautious interpretation. Further longitudinal studies are needed to better characterize QoL trajectories and identify subgroups requiring sustained intervention.

## Graphical abstract

**Figure:**
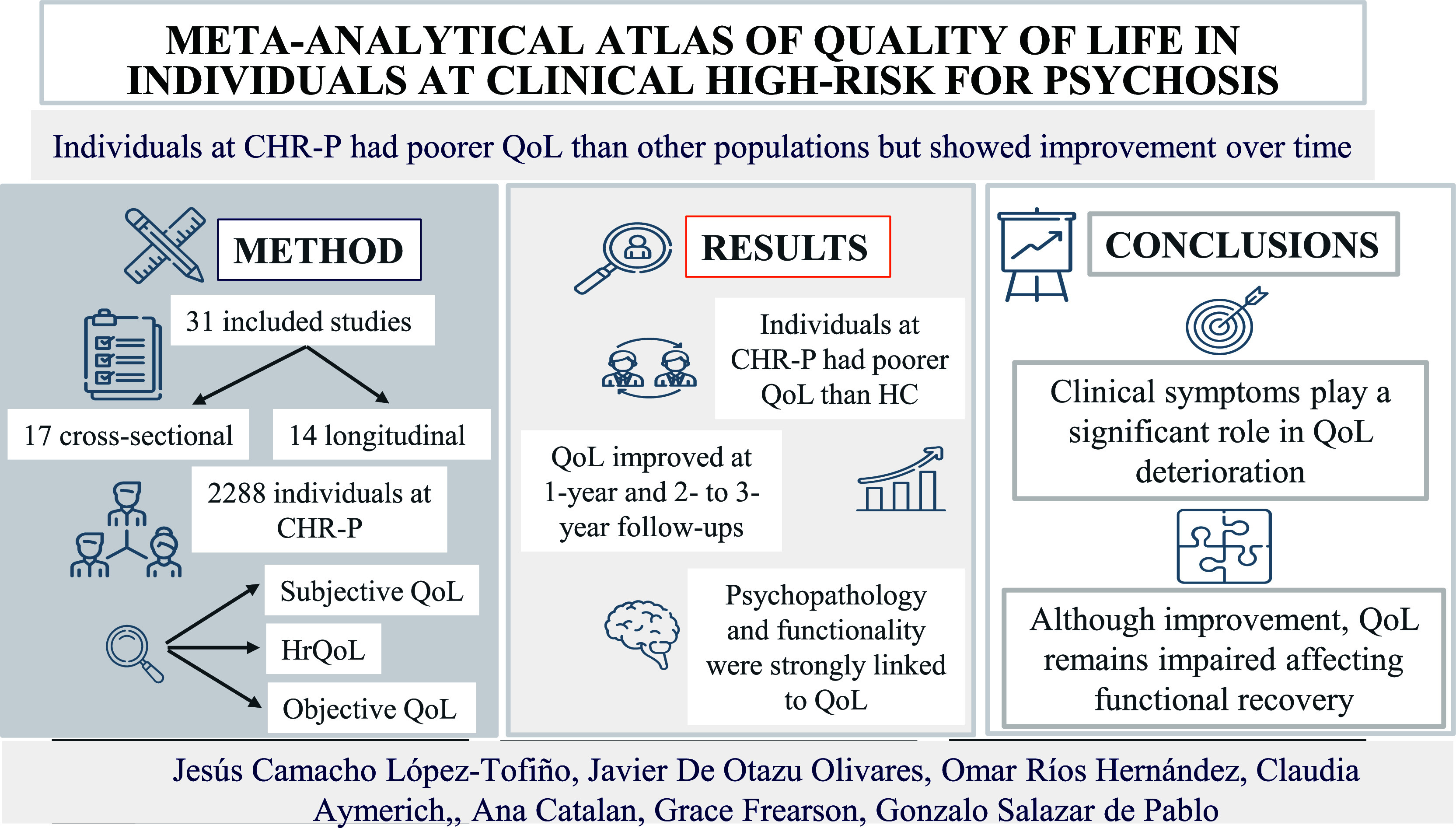

## Introduction

The clinical high-risk state for psychosis (CHR-P) paradigm was developed to identify people at high risk of developing a psychotic disorder over time [1]. This condition describes individuals characterized by attenuated psychotic symptoms, brief, limited, intermittent psychotic episodes, genetic vulnerability, or the presence of basic symptoms [2]. Individuals at CHR-P may progress to severe psychiatric disorders, primarily schizophrenia, with a progression rate of up to 25% over the following 2–3 years. However, the risk of transition continues to increase over the long term [3], and nearly one-third of individuals who do not develop psychosis experience persistent functional impairment [4–6].

Quality of Life (QoL) is a multidimensional construct encompassing life satisfaction, social functioning, and daily activities [7]. Although the term QoL can have varying definitions, most include physical, social, and psychological domains [8, 9]. For instance, according to the World Health Organization (WHO), health-related quality of life (HRQOL) is defined as individuals’ perceptions of their living conditions related to goals, expectations, standards, and concerns within different cultures and value systems [10]. Subjective QoL refers to individuals’ perceived well-being and satisfaction with life, while objective QoL involves externally observable indicators such as employment or housing status, which may not align with personal perception [11].

The assessment of QoL remains methodologically challenging in psychiatric research. Efforts to operationalize this construct have led to the development of numerous QoL instruments, including generic and disorder-specific measures, which differ in conceptual frameworks and assessment modalities, ranging from subjective self-report to clinician-rated scales. Despite this diversity, there is limited theoretical consensus on the definition of QoL, and it has often been treated as a secondary outcome rather than a core target for clinical decision-making. In this context, several authors have advocated for the complementary use of subjective and clinician-rated QoL instruments to capture a global and multidimensional representation of the construct, an approach that underpins the a priori focus on global QoL adopted in the present study.

Individuals at CHR-P consistently report lower levels of QoL than other clinical groups, often characterized by elevated distress, reduced autonomy, and impaired social integration [12–14]. The reduction in QoL has been linked to the severity of affective symptoms, particularly depression and anxiety [15, 16]. Notably, the presence of severe clinical symptoms at baseline also predicts poorer QoL outcomes of follow-up in this population [15]. Emerging studies have begun to explore the impact of early interventions on QoL in individuals at CHR-P. However, outcomes remain inconsistent, and persistence of subthreshold symptoms may contribute to ongoing impairment [17].

A previous meta-analysis [18] found that individuals at CHR-P had a large impairment in functioning and worse QoL than healthy controls, and notably, comparable QoL levels to those observed in patients with established psychosis. However, this meta-analysis was conducted 9 years ago and included only 4 studies. Furthermore, no meta-analytical evidence to date has examined the longitudinal trajectories of QoL in individuals at CHR-P.

Although interest in this area has grown in recent years, important gaps in the literature persist. A substantial proportion of studies continue to rely on cross-sectional designs, which limits our ability to draw conclusions about how QoL evolves over time. In addition, methodological heterogeneity – such as brief follow-up durations, the use of diverse QoL assessment tools, and small sample sizes – continues to pose challenges for synthesizing findings and advancing the field.

Based on the above, the aim of this study was (a) to evaluate the longitudinal trajectories of QoL in individuals at CHR-P; (b) to provide meta-analytical evidence comparing QoL in CHR-P subjects to healthy controls; and (c) to explore potential predictors or moderating variables influencing QoL in individuals at CHR-P.

## Methods

The protocol for this systematic review and meta-analysis was registered on PROSPERO (registration number: CRD42024560092). This study was reported according to the “Preferred Reporting Items for Systematic reviews and Meta-Analyses” (PRISMA, 2020) guidelines [19] and Meta-Analysis of Observational Studies in Epidemiology (MOOSE) [20] (eTable1–2), following EQUATOR Reporting Guidelines [21].

### Search strategy and selection criteria

A multi-step literature search was performed by three independent researchers (GF, JO, JC) in PubMed and Web of Science database (Clarivate Analytics), incorporating the Web of Science Core Collection, BIOSIS Citation Index, KCI-Korean Journal Database, MEDLINE, Russian Science Citation Index, and SciELO Citation Index, as well as Cochrane Central Register of Reviews and Ovid/PsycINFO databases, from inception until 11 May 2024. An updated search was conducted in databases on 27 May 2025.

The literature search was conducted using a single, predefined search strategy applied uniformly across all databases. The following search string was used. The following keywords were used: (“prodrom*” OR “ultra high risk” OR “clinical high risk” OR “high risk” OR “attenuat*” OR “APS” OR “brief limited” OR “brief intermittent” OR “BLIPS” OR “genetic high risk” OR “GRD” OR “at risk mental states” OR “risk of progression to first episode”) AND (“psychosis” OR “psychotic disorder” OR “schizophren*”) AND (“quality of life” OR “QOLS” OR “Satisfaction With Life Scale” OR “KIDSCREEN” OR “WHOQOL” OR “QOL” OR “MANSA” OR “QLS” OR “MSQOL”).

Articles identified were imported into EndNote for duplicate removal and were subsequently screened independently using the same management software by the same researchers, and those that were irrelevant were screened out. Title and abstract screening were conducted independently by at least two of the three researchers (GF, JO, and JC). The full texts of the remaining articles were assessed for eligibility, and decisions were made regarding their final inclusion in the review. Additionally, the references of systematic reviews and included articles were hand-searched for potentially relevant studies.

### Eligibility criteria

Inclusion criteria for the systematic review were (a) original individual studies; (b) participants were individuals at CHR-P (as per validated instruments and diagnostic criteria (eMethods1)); (c) cross-sectional or longitudinal, observational or intervention studies, (d) providing data on QoL according to validated measurement instruments (eMethods2); (e) published in English language. Exclusion criteria were (a) reviews, clinical cases, abstracts, conference proceedings, and study protocols, (b) studies conducted in individuals with established psychosis or otherwise not fulfilling CHR-P criteria, and (c) studies where QoL data were not reported. The systematic search did not retrieve any non-English records across the searched databases (n = 0); therefore, no studies were excluded based on language. When two or more studies from the same cohort were found, the largest and most recently published sample was selected for the meta-analyses. Disagreements in selection criteria were resolved through discussion and consensus with a senior researcher (CA).

### Data extraction and outcomes

Independent researchers (JC and ORH) extracted data from all included studies into an Excel spreadsheet. Data were cross-checked to ensure accuracy of the extraction, and disagreements were resolved through discussion between extractors and a senior researcher (GSP). Variables extracted included: age, sex, CHR-P subgroups [that is Attenuated Psychosis Symptoms (APS); Brief Intermittent Psychotic Symptoms (BLIPS)/Brief Limited Intermittent Psychotic Symptoms (BLIPS); Genetic Risk and Deterioration syndrome (GRD); Basic symptoms (BS)], instrument used to characterize CHR-P, type of comparison group (when it was applicable), sample size, country, study design, duration of follow-up, outcomes and study quality assessment (see below).

The primary outcome was the longitudinal change in QoL among individuals at CHR-P, assessed with validated instruments at baseline and follow-up. Cross-sectional comparisons with HC and FEP groups were considered secondary outcomes. Other outcome operationalizations (positive symptoms, negative symptoms, depressive symptoms, and functioning) are included in eTable3. Because the included studies used a wide range of QoL instruments, we applied a systematic harmonization procedure before conducting the meta-analyses. First, for each instrument, score directionality was standardized so that higher scores consistently reflected poorer QoL, reversing scales when necessary. Second, when studies reported multiple QoL outcomes, the total QoL score was always prioritized over subscales, and if several total scores were available, we extracted the authors’ primary or most global QoL measure. Third, because QoL instruments differ in maximum scores, ranges, and distributions, all outcomes were converted to a common metric (Hedges’g) using means, SDs, and sample sizes. This ensured statistical comparability across studies. In addition, all included studies were systematically screened for domain-level QoL outcomes. Although several studies reported domain-specific scores and, in some cases, provided domain-level SD, reporting was highly heterogeneous across instruments and scoring metrics. As a result, domain-level quantitative synthesis or sensitivity analyses were not methodologically feasible (eTable 10).

### Risk of bias (quality) assessment

The quality of the included studies was evaluated using a modified version of the Newcastle–Ottawa Scale (NOS) for cohort and cross-sectional studies, which has been frequently employed in systematic reviews and meta-analyses in the field [22, 23]. Studies were awarded a maximum of eight points according to their representativeness, exposure, outcomes, follow-up period, and losses to follow-up. In addition to total scores, domain-level judgments were derived by mapping individual NOS items onto major sources of bias: selection bias, confounding, and outcome-related bias (including detection and attrition). Individual study scores and domain-level assessments are reported in Supplementary Material (eTable4–5).

### Qualitative synthesis

We conducted a narrative synthesis of the findings from the included studies. The primary focus was on longitudinal changes in QoL among individuals at CHR-P, complemented by secondary analyses of cross-sectional differences (CHR-P vs HC and CHR-P vs FEP). Results were organized into two main sections: (1) cross-sectional analyses, with particular attention given to comparisons between CHR-P versus HC and CHR-P versus FEP groups to better understand differences in QoL across stages of illness, and (2) longitudinal analyses, examining changes in QoL over time within individuals at CHR-P. This dual structure allowed us to capture both snapshot comparisons and the progression of QoL changes over time.

### Quantitative synthesis

Meta-analyses primarily focused on the longitudinal change in QoL among CHR-P individuals across follow-up timepoints (12, 24, and 36 months when available). Secondary analyses examined cross-sectional comparisons between CHR-P and HC groups. The comparison of effect sizes in each group was calculated as the Hedges’ g. A positive Hedge’s g indicates that the CHR-P population has poorer QoL compared with the HC group. Hedges’ g = 0.2 was interpreted as a small effect size, Hedges’ g = 0.5 as a medium effect size, and Hedges’ g = 0.8 as a large effect size [24, 25]. Effect sizes were calculated using the means, standard deviations (SDs), and sample sizes for the outcome of interest for each sample. For each outcome, data were extracted at baseline and at different points of follow-up (12 months, 24 months, and 36 months) when it was provided. Meta-analyses were conducted using random-effects models to account for between-study heterogeneity. The primary analyses employed the DerSimonian and Laird method for estimation of the between-study variance, as implemented in Comprehensive Meta-Analysis Software, version 3 (Biostat, Inc) [26]. Given the high levels of heterogeneity and the relatively small number of studies in some analyses, sensitivity analyses were performed by re-estimating all main models using a restricted maximum likelihood (REML) estimator. Results were compared across estimators to assess the robustness of pooled effect sizes to the choice of variance estimation method. This choice was further supported by the results of the Q statistic and the *I*^2^ index, which confirmed substantial between-study heterogeneity [27]. Potential publication bias and small study effects were further explored using funnel plots and performing Egger’s test [27]. Given the small number of studies contributing to several analyses and the very high between-study heterogeneity, results from Egger’s test were interpreted cautiously, as the test is known to be underpowered under these conditions. The feasibility of longitudinal analyses stratified by QoL instrument family is reported in eTable 11.

Meta-regression analyses were performed when at least six studies per outcome were available, ensuring a minimum data threshold for reliable estimates in line with recommendations for small-sample meta-analyses [28]. We investigated the influence of the following variables: (a) mean age, (b) sex (% female), (c) level of functioning, (d) positive symptoms severity, (e) negative symptoms severity, and (f) quality of the included studies (NOS total score). Subgroup analyses were performed, including (a) continent (Europe vs other), (b) type of psychometric instrument (CAARMS vs SIPS vs other), and (c) type of QoL scale used (QlS vs other). Because QlS-based instruments are clinician-rated, whereas non-QlS instruments are predominantly self-report measures, this subgrouping operationalized comparisons between clinician-rated and self-reported assessments of QoL. The significance level was set at p < 0.05, two-sided. To address potential scale-related heterogeneity and improve the interpretation of large longitudinal standardized effects, we explored the feasibility of instrument-stratified longitudinal analyses by QoL instrument family (g. e., QLS-based, WHOQOL-based, AQoL-based, MSQoL-based, and HRQoL-based instruments). Where at least two studies were available within an instrument family, results were examined descriptively; however, due to the limited number of studies per family, formal pooled estimates were only computed when statistically appropriate. In most cases, instrument-stratified analyses were summarized descriptively rather than meta-analytically.

## Results

### Search results

**Figure 1.**
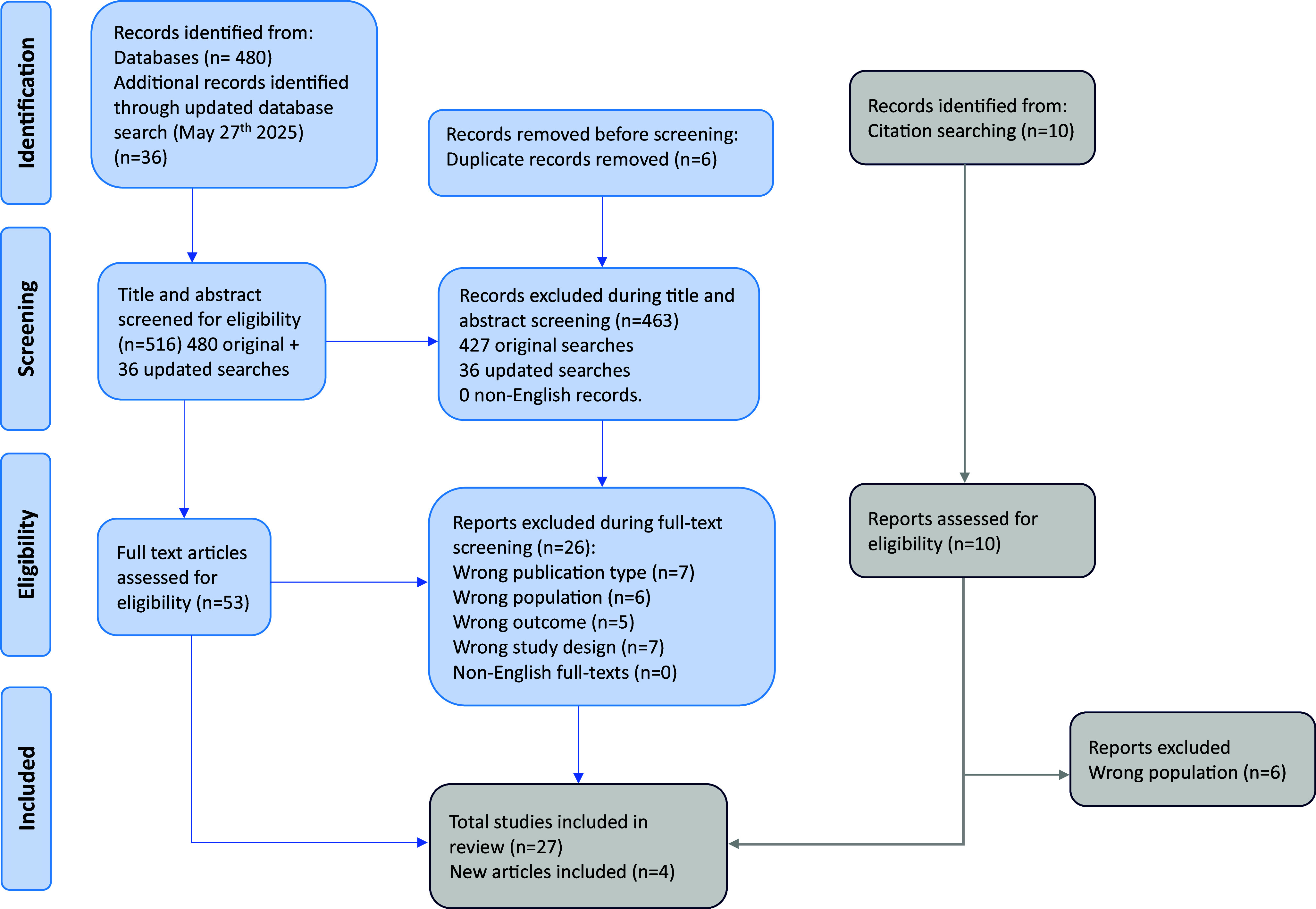
PRISMA 2020 flow diagram.

The literature search yielded 480 citations through electronic databases, which were screened for eligibility. An updated search identified 36 additional records, resulting in a total of 516 citations screened. Of these, 53 articles were assessed in full text, and after excluding those not meeting the inclusion criteria, 31 studies were included in the final systematic review and meta-analysis database (Figure 1). Seventeen were cross-sectional (54.84%) [4, 6, 9, 11–13, 16, 29–38] and 14 longitudinal (45.16%) [5, 15, 39–50].

### Study characteristics

The overall database comprised 2,288 CHR-P individuals. The mean age and SD of the sample were 21.47 ± 4.2 years, and 48.85% were females. Fifteen (48.39%) studies were conducted in Europe, two (6.45%) in North America, eight (25.81%) in Asia, and six (19.35%) in Oceania. The mean follow-up in the meta-analyzed longitudinal included studies was 15.9 months with a SD of 16.87 and a range of 1.5–72 months (eTable8). A summary table of all included articles is available in Table 1.

**Table 1.** Characteristics of all the included studies

First author, year	Country	Design	CHR-P sample size	CHR-P subgroups	Age: mean, (SD), range	Sex: % female	CHR-P assessment tools	QoL assessment tools	Total duration (months)	NOS score
Addington, 2008 [4]	Canada	Cross-sectional	86	n/a	19.4 (4.5)	43%	SIPS	QLS (role)	n/a	7
Bechdolf, 2005 [12]	Germany	Cross-sectional	45	n/a	25.7 (5.0)	31.1%	ERI (IRAOS)	MSQoL	n/a	6
Fallah Zadeh, 2023 [29]	Iran	Cross-sectional	40	n/a	27.5 (5.3)	70%	SIPS	QLS	n/a	6
Francey, 2005 [5]	Australia	Longitudinal	20	42.9% APS, 30% BLIPS, 27.2% GRD*	20.9	40%	BPRS	QLS	n/a	7
Glenthoj, 2020a [30]	Denmark	Longitudinal	146	76% APS, 1.4% GRD, 20.5% APS + GRD, 2.1% APS + BLIPS	23.9 (4.2)	58.2%	CAARMS	AQoL (8D)	12	5
Glenthoj, 2020b [39]	Denmark	Cross sectional	59	75.9% APS, 0.8% GRD, 21.8% APS+ GRD, 2.3% APS + BLIPS	23.5 (4.3)	59.3%	CAARMS	AQoL	n/a	6
Grano, 2014 [9]	Finland	Cross-sectional	66	n/a	15.6 (2.1)	69.7%	PRODS/SIPS	HRQoL 16D	n/a	6
Grano, 2011 [11]	Finland	Cross-sectional	43	n/a	14.7 (1.7)	65%	PRODS/SIPS	HRQoL 16D	n/a	6
Grano, 2009 [40]	Finland	Longitudinal	28	n/a	14.5, 12–18	64%	PRODS/SIPS	HRQoL 16D	7	3
Hauser, 2009 [41]	Germany	Longitudinal	16	87.5% APS, 6.25% BLIPS, 6.25% BS	26 (4.9)	25%	CAARMS	MSQoL	1.5	4
Heinze, 2018 [42]	UK	Longitudinal	14	n/a	20.8 (2.6)	64.2%	CAARMS	WHOQOL-BREF	12	5
Hui, 2013 [31]	UK	Cross-sectional	60	100% APS, 11.7% GRD	20.2 (2.9)	48.3%	CAARMS	MANSA	n/a	6
Kim, 2019 [33]	South Korea	Cross-sectional	60	71.6% APS, 1.6% BLIPS, 8.3% APS + BLIPS, 18.3% APS + GRDS	20.3 (3.5)	43.3%	SIPS	QLS	24	7
Kim, 2013 [32]	South Korea	Cross-sectional	60	n/a	20.2 (2.9)	48.3%	SIPS	QLS	n/a	7
Lin, 2013 [43]	Australia	Longitudinal	228	62.3% APS, 4.4% BLIPS, 13.6% GRD, 3.9% APS + BLIPS, 12.8% combined categories**	25.7 (4.9)	59.2%	CAARMS	QLS	n/a	5
Mao, 2023 [11]	China	Cross-sectional	59	n/a	21.7 (5.4)	40.6%	SIPS	WHOQOL-BREF	n/a	6
McFarlane, 2015 [44]	USA	Interventional (quasi-experimental)	205	n/a	16.4 (3.3)	43%	SIPS	QLS	24	7
McGorry, 2023 [46]	Australia	Interventional (RCT)	115	n/a	18.1 [3]	60.2%	CAARMS	AQoL	12	8
McGorry, 2013 [45]	Australia	Interventional (RCT)	115	n/a	17.7 (3.1), 12.5–25	57.9%	CAARMS	QLS	12	7
Michel, 2019 [34]	Switzerland	Cross-sectional	370	7.5%APS, 0.1% BLIPS, 9.8% BS	30.7 (7.6), 15.3–41.5	46%	SIPS	BMLSS	n/a	6
Morita, 2014 [47]	Japan	Longitudinal	46	100% APS, 19.6% BLIPS, 45.7% GRD	23.5 (6.6)	71.7%	SIPS	WHOQOL–26	12	4
Morrison, 2012 [48]	UK	Interventional (RCT)	195	n/a	20.7 (4.3)	37.5%	CAARMS	MANSA	24	6
Nitka, 2016 [38]	Germany	Cross-sectional	27	n/a	15.4 (1.1), 14–18	48.1%	SIPS	HRQoL	n/a	4
Ohmuro, 2007 [37]	Japan	Cross-sectional	104	81% APS, 2% BLIPS, 2% STF, 14% APS + STF, 2% APS + BLIPS	19.9 (4.2)	63%	CAARMS	WHOQOL-BREF	n/a	7
Ortega, 2018 [35]	Spain	Cross-sectional	55	n/a	22.4 (4.4)	29.1%	CAARMS	EQ–5D	n/a	6
Ruhrmann, 2008 [36]	Germany	Cross-sectional	215	n/a	25.9 [6]	37.7%	ERI (IRAOS)	MSQoL	n/a	6
Stain, 2016 [49]	Australia	Interventional (RCT)	57	81% APS, 7% BLIPS, 19% GRD	16.3 (2.9)	59.7%	CAARMS	QLS	12	6
Svirskis, 2007 [13]	Finland	Cross-sectional	39	n/a	[14-40]	67.8%	SIPS	QLS	n/a	6
Takahashi, 2017 [6]	Japan	Cross-sectional	33	100% APS	17.7 [3]	45.5%	CAARMS-J	QLS	<12	5
Usui, 2022 [15]	Japan	Longitudinal	22	63.6% APS, 4.5% BLIPS, 9.1% GRDS, 22.7% APS + GRDS	21.1 [4]	54.5%	SIPS	WHOQOL	24	4
Velthorst, 2013 [50]	Australia	Longitudinal	157	80.9% APS, 13.4% BLIPS, 24.2% GRD	19.3 (3.3)	41.4%	SIPS	QLS	72	5

Abbreviations: APS, Attenuated Psychosis Syndrome; AQoL, Assessment of Quality of Life; BDI-II, Beck Depression Inventory-II; BLIPS, Brief Limited Intermittent Psychotic Symptoms; BMLSS, Brief Multidimensional Life Satisfaction Scale; BPRS, Brief Psychiatric Rating Scale; BS, Basic Symptoms; CAARMS, Comprehensive Assessment of At Risk Mental States; CHR-P, Clinical High Risk for Psychosis; EQ-5D, Euroqol Five Dimensions; ERI (IRAOS), Early Recognition Inventory (Interview for the Retrospective Assessment of the Onset and course of Schizophrenia and other psychoses; FEP, First Episode of Psychosis; FHR, Familial High Risk; FU, Follow-Up; GAF, Global Assessment of Functioning; GRDS, Genetic Risk and Deterioration Syndrome; H, Healthy Controls; HRQoL, Health Related Quality Of Life; MADRS, Montgomery-Asberg Depression Rating Scale; MANSA, Manchester Short Assessment of Quality of Life; MSQoL, Modular System for Quality of Life; QoL, Quality of Life; QLS, Quality of Life Scale; NOS, Newcastle–Ottawa Scale; PANSS, Positive and Negative Syndrome Scale; PRODS, Prodromal Risk of Disorder Screen; SANS, Scale for the Assessment of Negative Symptoms; SASS, Social Adaptation Self-Evaluation Scale; SCoRS, Schizophrenia Cognition Rating Scale; SFS, Social Functioning Scale; SIPS, Structured Interview of Psychosis-risk Syndromes; SOFAS, Social and Occupational Functioning Assessment Scale; SOPS, Scale Of Prodromal Symptoms; STAI, State Trait Anxiety Inventory; STF, Self-Experienced Transition Features; WHOQOL, World Health Organization for Quality Of Life.

*Note*: *Percentages may exceed 100% as cases can belong to multiple categories**Combined categories included individuals with both trait vulnerability+APS (0.1%), trait vulnerability+BLIPS (0.9%), trait vulnerability+APS + BLIPS (1.8%). Overlapping cohorts and handling of duplicate publications are detailed in Supplementary Table S1.

### Quality assessment and publication bias

The quality of the included studies ranged from 1 to 8, with a median of 6 and a mean of 5.8 (s.d. = 1.13) (eTable5). Funnel plot inspection suggested a potential asymmetry in the analysis comparing CHR-P and HC groups (eFigure 1). However, Egger’s test was not statistically significant (t = 1.93, p = 0.085). Publication bias was not detected in the Egger’s test or funnel plot inspection (all p > 0.05) for any of the other evaluated comparisons (eFigure 2 and eFigure 3, eTable6).

### Qualitative synthesis

To measure QoL, 12 studies (38.7%) used the QLS (Quality of Life Scale) [4–6, 13, 29, 32, 33, 43–45, 49, 50], five (16.1%) the WHOQOL-BREF (World Health Organization Quality of Life-BREF) scale [11, 15, 37, 42, 47], three studies (9.7%) the HRQoL 16D (Health Related Quality of Life 16 Dimensions) scale [9, 16, 40] (only 1 study adopted another version, KIDSCREEN-27 HRQoL [38]), three studies (9.7%) the AQoL (Assessment of Quality of Life) scale [30, 39, 46], two studies (6.5%) the MANSA (Manchester Short Assessment of Quality of Life) scale [31, 48], three studies (9.7%) the MSQoL (Modular System for Quality of Life scale) [12, 36, 41], one study (3.2%) the EQ-5D (Euro Quality of life five dimensions) scale [35], and one study (3.2%) the BMLSS scale [34].

All of them found that individuals at CHR-P presented poorer QoL compared to HC subjects. Six cross-sectional studies also included FEP groups for comparison with CHR-P individuals [4, 5, 11, 12, 33, 37]. Among these, Addington, Penn [4], and Bechdolf, Pukrop [12] reported similar QoL results between both groups, with no significant differences observed. However, the remaining four studies found lower QoL in individuals at CHR-P compared to FEP (p < 0.05). An exception was observed in the study by Francey and Jackson [5], where individuals at CHR-P reported better QoL than those in the FEP group (p < 0.05).

In relation to psychopathological variables, eight studies [5, 11, 13, 30, 32, 33, 43, 47] found a negative correlation between QoL and negative symptoms, with reported correlation coefficients ranging from r = −0.79 to r = −0.29, p < 0.05, implying that poorer QoL was associated with greater severity of negative symptoms. In one study [47], it was found that patients who dropped out of follow-up had less severe negative symptoms and better QoL compared to those individuals at CHR-P who continued longitudinal follow-up. Only one study [37] did not find a significant relationship between the severity of negative symptoms and QoL.

Additionally, several studies have found that higher severity of depressive symptoms was associated with lower QoL, with reported correlation coefficients ranging from r = −0.56 to −0.32, p < 0.05 [11, 13, 15, 16, 32, 37].

In terms of functionality, measured with different instruments across studies (Global Assessment of Functioning [GAF], the Social and Occupational Functioning Assessment Scale [SOFAS] and the Social Adaptation Self-evaluation Scale [SASS]), five studies [13, 16, 31, 35, 43] found a positively significant correlation between QoL and level of functioning in individuals at CHR-P, with reported correlation coefficients ranging from r = 0.35 to r = 0.84, p < 0.05.

Fourteen studies were longitudinal [5, 15, 39–50]. Of these studies, the majority conducted a minimum longitudinal follow-up in the QoL variable of 12 months, with a follow-up extended to 24 months in three studies [15, 44, 48]. Four studies [5, 15, 42, 44] compared CHR-P with other groups. Heinze, Lin [42] compared individuals at CHR-P with individuals not at CHR-P (individuals with no significant psychotic experiences or individuals at risk without functional decline), and they found lower QoL in CHR-P participants. Three studies compared CHR-P with FEP individuals. McFarlane, Levin [44] did not find differences between both groups; however, patients at CHR-P showed poorer QoL than FEP individuals in two studies [5] [15].

The rest studies conducted longitudinal follow-up only on the group of CHR-P patients, and the majority found better QoL at follow-up for these individuals [39–41, 45–47, 49]. Morrison, French [48] et al. did not find significant differences between baseline and follow-up data. Velthorst, Nelson [50] found that the transition rate to develop psychosis in the deteriorating QoL group was significantly higher than the transition rates in groups with better QoL scores. A summary of the key findings from the studies reviewed is presented in Table 2 to facilitate comparison across studies and highlight relevant patterns related to QoL in CHR-P populations.

**Table 2.** Key findings summary

Study (Author, Year)	Key findings
Addington, 2008 [4]	Individuals with CHR-P and FEP groups performed similarly on QLS, significantly poorer than HC and significantly better than chronic schizophrenic patients. QLS and SFS scores were significantly associated in individuals with CHR-P (r = 0.4, p < 0.001), specifically with the employment sub-score of SFS, compared to HC.
Bechdolf, 2005 [12]	Compared to HC, individuals with CHR-P obtained significantly lower QoL scores in five of seven domains (physical health, vitality, psychosocial, affective, and general QoL). Higher scores in PANNS positive correlated significantly with lower scores in several QoL domains (psychosocial, spare time, affective, and general QoL) (p0.05, p0.01). Employment or being in training was significantly associated with lower psychosocial and general QoL scores.
Fallah Zadeh, 2023 [29]	Individuals with CHR-P had poorer QoL results compared to FHR (75.17).
Francey, 2005 [5]	Individuals with CHR-P had poorer QoL compared to the FEP group (F (2.98) =3.27, p = .04 CHR > FEP). Within individuals with CHR-P subgroups, CHR-P obtained poorer QoL than CHR-NP. Individuals with CHR-P had higher scores on SANS negative symptoms.
Glenthoj, 2020a [30]	The longitudinal outcomes in this study revealed a better QoL at FU compared to baseline (0.53 vs 0.44). QoL was inversely correlated with negative symptoms, specifically with avolition (−0.324, p < 0.001).
Glenthoj, 2020b [39]	Individuals with CHR-P experiencing cognitive deficits had lower QoL and functioning compared to individuals with CHR-P without cognitive disturbances.
Grano, 2014	Individuals with CHR-P obtained poorer QoL scores compared to HC (0.872 vs 0.795, p < 0.001). Specifically, significant differences were found in vitality, distress, physical appearance, school and hobbies, friends, mental function, and depression.
Grano, 2011 [16]	Individuals with CHR-P had poorer scores in QoL, functioning, and depressive symptoms compared to HC.
Grano, 2009	The longitudinal outcomes in this study revealed a better QoL at FU compared to baseline (10.9).
Hauser, 2009 [41]	After receiving psychoeducation, individuals with CHR-P improved significantly in QoL compared to baseline (z = −2.2276, p = 0.023). Specifically, three of seven areas improved considerably: vitality (z = −2.249, p = 0.204), free-time (z = −2.554, p = 0.011), and affective QoL (z = −2.272, p = 0.023).
Heinze, 2018 [42]	Individuals with CHR-P showed lower QoL than non-CHR over time (F (1, 160.54) =7.68, p = 0.006).
Hui, 2013 [31]	Individuals with CHR-P reported poorer QoL (3.8), GAF symptoms (45.4), and disability (48.6) than HC.
Kim, 2019 [33]	Individuals with CHR-P showed lower QoL than FEP (70.1 vs 83.54, p < 0.001). 38% of QLS score variance was explained by SANS score, social cognitive bias, reflective self, and neurocognition.
Kim, 2013 [32]	Individuals with CHR-P had poorer QoL than HC (55.4). QoL was significantly correlated with general (r = −0.34, p < 0.01) and negative symptoms of SIPS (r = −0.58, p < 0.001), and depressive symptoms of MADRS (r = −0.35, p < 0.01).
Lin, 2013 [43]	QoL was positively associated with functioning, and negatively associated with negative symptoms (and introverted anhedonia.
Mao, 2023 [11]	Compared to HC and FEP groups, QoL scores revealed that Individuals with CHR-P had lower scores in total WHOQOL-BREF, and psychological health QoL was associated with severity of negative symptoms (r = −0.29, p = 0.03) and depressive symptoms (r = −0.33, p = 0.01). Unemployment also had a negative influence on QoL (t = −2.97, p = 0.005). Otherwise, QoL and physical health improved with age.
McFarlane, 2015 [44]	Individuals with CHR-P improved QoL significantly from baseline (t = 7.508, df = 127, p < 0.0001).
McGorry, 2023 [46]	Individuals with CHR-P improved QoL at 6 months FU (F = 0.07, p = 0.6) and at 12 months FU (F = –0.10, p = 0.54) in both treatment conditions.
McGorry, 2013 [45]	Individuals with CHR-P improved QoL at 12 months FU in all groups. Change, Mean SD = (10.618.6, p = 0.200).
Michel, 2019 [34]	Lower QoL was significantly associated with any affective disorder.
Morita, 2014 [47]	Individuals with CHR-P improved QoL at 12 m FU (62.8, 58.4). Otherwise, individuals with CHR-P that withdraw of the study had fewer negative and general symptoms and a higher QoL.
Morrison, 2012 [48]	There were no significant differences between means comparing at baseline and at FU (2.24, −0.60 to 5.08; p = 0.12).
Nitka, 2016 [38]	Individuals with CHR-P reached significantly poorer scores compared to HC, specifically in physical well-being (t [55] = − 2.3, p < 0.025), psychological well-being t [55]= − 2.03, p = 0.047), and school environment (t [55]= − 2.41, p = 0.021). They found that lower scores in the scale school environment explained at-risk status for psychosis (OR = 1.16, 95% CI = 1.01–1.30, p = 0.03).
Ohmuro, 2007	Individuals with CHR-P showed poorer QoL than FEP patients, with physical health (35.8 and psychological health (domains reaching significant differences. However, the significance of these differences disappeared when PANSS depression or BDI-II was controlled for (physical health: P = 0.23 and 0.90; psychological health: p = 0.09 AND 0.53, respectively). In both groups, higher severity of depressive symptoms was associated with lower QoL. No significant correlations between the severity of negative symptoms and QoL were found.
Ortega, 2018	Individuals with CHR-P reported a poorer QoL than HC (0.63 vs 0.93, p < 0.001), and also higher stress and lower social adaptation. QoL was negatively associated with perceived stress (r = −0.71, p < 0.001), and positively associated with functioning measured by SASS (r = 0.35, p < 0.05).
Ruhrmann, 2008 [36]	Demographics as age, regular occupation, and having a steady partner, were associated significantly with higher QoL (r = 0.29, p = 0.030; t [54] = − 2.13, p = 0.038; t [50]= − 2.46, p = 0.017, respectively). Individuals with CHR-P showed a significantly lower QoL than HC (43.5 vs 80, p). Depression was a significant predictor in all QoL areas.
Stain, 2016 [49]	Individuals with CHR-P QoL scores at FU were higher than baseline, indicating an improvement in QoL (26.07 vs 31.57).
Svirskis, 2007 [13]	Individuals with CHR-P had a poorer QoL score than HC (84.7). Living alone, having low education, and being a treatment-seeker were associated with lower QoL and functioning.
Takahashi, 2017 [6]	Individuals with CHR-P showed poorer QoL than HC (57.08). In individuals with CHR-P group, QoL was significantly associated with SOFAS (r = 0.81, p < 0.001), and negatively associated with BDI-II (r = −0.65, p < 0.001), SCoRS (r = −0.079, p < 0.001), PANSS negative and general (r = −0.58, r = −0.68, p < 0.001) and STAI trait and state (r = −0.62, r = −0.58, p < 0.001).
Usui, 2022 [15]	Individuals with CHR-P showed lower scores in physical (F = 4.98, p < 0.05) and psychological health (F = 5.99, p < 0.05) QoL subscales than FEP. Longitudinally, QoL improved specifically in physical health (F = 21.56, p < 0.001), psychological health (F = 24.78, p < 0.001), and environment (F = 24.78, p < 0.001). Higher anxiety/depression at baseline was significantly associated with poorer QoL in social relationships and the environment. Additionally, improvement of anxiety/depression was significantly related to better physical health, and improvement of disorganized thoughts was significantly correlated with improvement of QoL in psychological health and social relationships.
Velthorst, 2013 [50]	Individuals with CHR-P with deteriorating QLS had greater transition rates than the improving QLS group. In this study, individuals with CHR-P who had a similar QLS score at baseline and at FU and who deteriorated had a higher likelihood of transitioning to psychosis than participants with an improvement in QLS score. They also found that Low and Improving QLS groups had similar baseline negative symptoms scores, both significantly higher in severity than the negative symptoms at High QLS group.

### Quantitative synthesis

#### Quality of life comparing CHR-P versus HC

**Figure 2.**
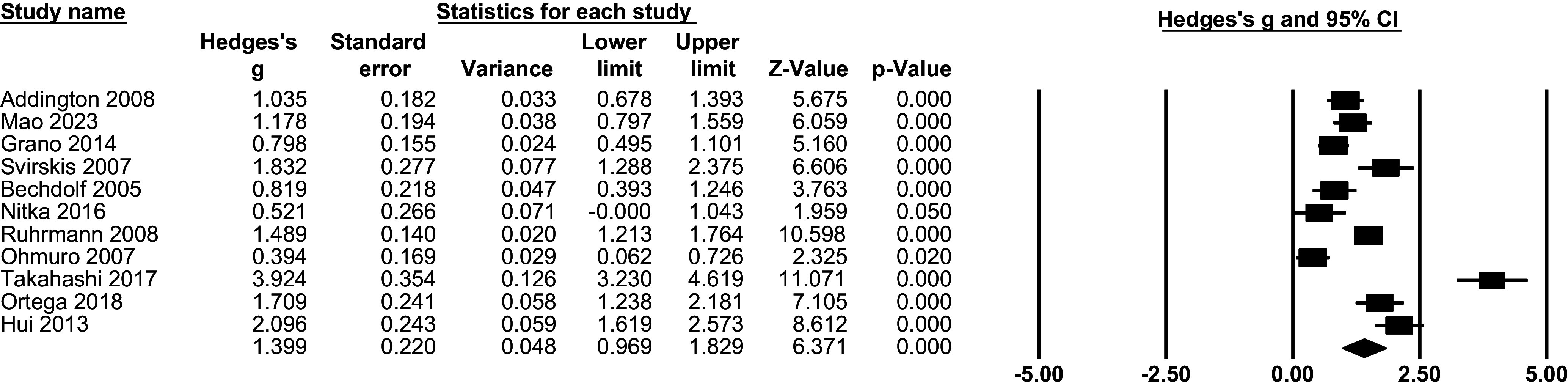
CHR-P vs HC: Quality of life: forest plots of meta-analysis.

Pooled Hedge’s g for QoL in the CHR-P compared with the HC group was 1.39 (95% CI 0.97; 1.83, p < 0.001, k = 11), implying a significantly poorer QoL among the CHR-P population (Figure 2).

Heterogeneity for this comparison was statistically significant (**I**^**2**^ = 92.09%, p < 0.001). Subgroup analyses grouped studies based on characteristics, but no significant differences were found in terms of continent (Europe vs other), scale used (QlS vs other), or instrument (CAARMS vs SIPS vs other) (all p > .05, eTable7). However, point estimates suggested descriptively larger effects in QlS-based studies compared with non-QlS instruments (eTable7), despite overlapping confidence intervals and non-significant subgroup tests. Additionally, meta-regressions did not reveal significant moderating effects of age, % females, level of functioning, positive symptoms, negative symptoms, or quality assessment (all p > .05). These analyses were based on a limited number of studies (k = 6–11, depending on the moderator examined; eTable9), which reduced statistical power and limits the interpretability of moderator effects.

#### Longitudinal changes of QoL over time

**Figure 3.**
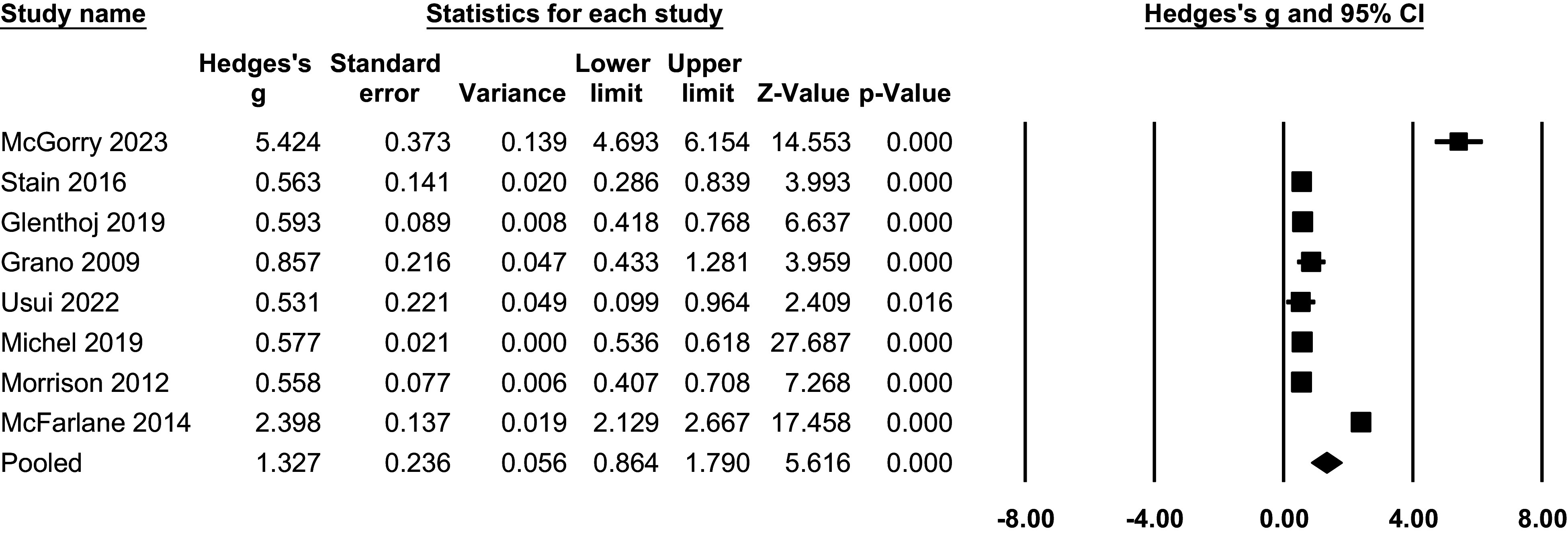
Longitudinal progression of QoL at 1-year follow-up: forest plot of meta-analysis.

**Figure 4.**
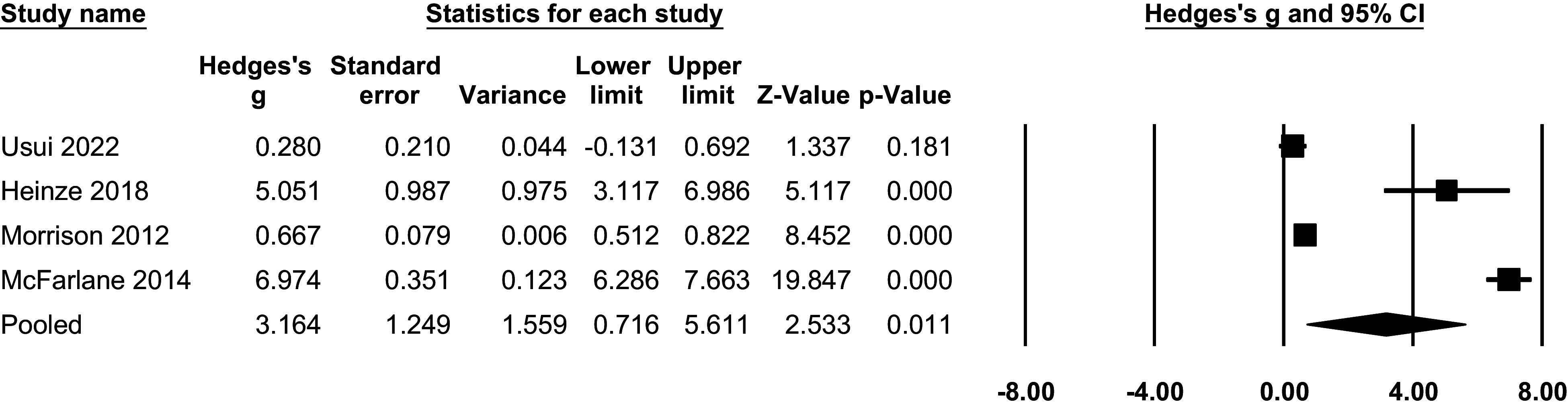
Longitudinal progression of QoL at 2–3-year follow-up: forest plot of meta-analysis.

Meta-analysis findings indicated that individuals at CHR-P state showed improved QoL at both follow-ups. At 1 year, QoL significantly improved compared to baseline (Hedges’g = 1.33, 95% CI = 0.86 to 1.79, P < 0.001, n = 8; Figure 3). At 2 to 3 years, larger pooled effect sizes were observed; however, these estimates were highly heterogeneous and largely driven by a single study, and should therefore be interpreted cautiously (Hedges’g = 3.16, 95% CI = 0.72 to 5.61, P < 0.05, n = 4; Figure 4).

Heterogeneity was also statistically significant for the longitudinal analyses (**I**^**2**^ = 97.72%, p < 0.001). Subgroup analyses revealed that participants from studies conducted in Europe had poorer QoL compared to participants from other continents at 1-year follow-up (Q = 4.096, p = 0.043). No significant differences were observed for other sub-analyses (all p > .05, eTable8). Similarly, meta-regressions did not show significant moderating effects for the variables tested (all p > .05, eTable9).

Leave-one-out sensitivity analyses indicated that pooled estimates at 1-year follow-up were generally robust. In contrast, the long-term (2–3 years) pooled effect was largely driven by a single study reporting a markedly larger effect size (eFigure4–6). This indicates that the long-term estimate is highly sensitive to individual studies and should therefore be interpreted with caution [44]. Stratified analyses by QoL instrument family did not materially alter the overall pattern of results, although long-term estimates remained unstable due to the small number of contributing studies.

## Discussion

To our knowledge, this is the first systematic review and meta-analysis of individuals at CHR-P to evaluate longitudinal changes in QoL, including cross-sectional QoL data too. Two main findings were identified: (1) Individuals at CHR-P exhibited poorer QoL compared to HC and FEP individuals; and (2) longitudinal analyses suggested a potential improvement in QoL among individuals at CHR-P when measured at least 12 months apart. This meta-analysis is based on 2288 CHR-P individuals with a mean age of 21.47 years and a slightly higher frequency of males (51.15%), aligning with the typical sociodemographic profile of this group.

A key finding is that individuals at CHR-P exhibited poorer QoL than the HC group, aligning with previous meta-analytic findings [18] that showed QoL in individuals at CHR-P is comparable to patients with psychosis but significantly worse than HC. This pattern is consistent with evidence showing that even subthreshold psychotic experiences – as observed in CHR-P states – are associated with reductions in QoL. Although CHR-P individuals do not meet full criteria for psychotic disorders, attenuated symptoms, affective dysregulation, and emerging functional difficulties can contribute to perceived stigma, social withdrawal, and reduced well-being [51]. These mechanisms may partially overlap with those described in early psychosis, thereby helping to explain the markedly lower QoL observed in CHR-P populations [52, 53]. Additionally, previous findings suggest that psychopathology, particularly negative and depressive symptoms, is strongly linked to reduced QoL in psychosis, as observed in studies of schizophrenia and FEP samples [54–56]. Although subgroup analyses by QoL instrument were not statistically significant, QlS-based studies showed descriptively larger effect sizes than non-QlS instruments. This pattern should be interpreted cautiously, given overlapping confidence intervals and limited statistical power, but may reflect greater sensitivity of the QlS developed for schizophrenia-spectrum conditions to the functional and social impairments already present in the CHR-P phenotype.

The extremely high between-study heterogeneity observed across analyses (**I**^**2**^ > 90%) likely reflects a combination of clinical, methodological, and measurement-related sources that could not be fully captured through study-level meta-regression. First, QoL is a multidimensional construct assessed using instruments that differ substantially in conceptual focus (subjective self-report vs clinician-rated), domain coverage, and scaling properties. Second, included studies varied widely in design (cross-sectional vs longitudinal), follow-up duration, and clinical context, including recruitment through early intervention services versus naturalistic samples. Third, some cohorts received preventive psychological or pharmacological interventions, whereas others followed monitoring-only approaches, introducing additional variability in QoL trajectories. Finally, methodological factors such as selective attrition, regression to the mean, and response-shift effects may have further inflated heterogeneity, particularly in longitudinal analyses. Together, these factors help explain why conventional meta-regression analyses did not identify statistically significant moderators despite substantial between-study variability.

Our results are consistent with previous findings in individuals at CHR-P, where poorer QoL was associated with greater severity of negative and depressive symptoms. Previous work has shown that depressive symptoms are a strong predictor of lower QoL in various populations [57–59], and a moderate relationship has been reported between symptom reduction and QoL improvement, as suggested by a meta-analysis [56]. While negative symptoms and functional impairments have been linked to QoL in individuals at CHR-P [32, 60], these factors alone do not fully explain the deterioration observed in this population [6]. Other risk factors, such as perinatal issues and experiences of abuse or emotional trauma during childhood and adolescence, have been identified as contributors to increased vulnerability in this group [23, 61]. These early-life vulnerabilities, along with deficits in academic and social functioning, have been closely associated with poorer neurocognitive performance [62] and may help explain the long-term deterioration in QoL observed in individuals at CHR-P.

In addition, recent evidence suggests that the relationship between QoL and core symptoms may be bidirectional. A meta-analysis conducted in adolescents with early-onset psychosis and individuals at CHR-P found that baseline negative symptoms predicted poorer functioning at both 1- and 2- year follow-up [63]. However, greater severity of negative symptoms was also observed in those with lower levels of social support and co-occurring depressive symptoms – both of which are strongly associated with reduced QoL. These findings point to a potential feedback loop in which poor QoL may contribute to the persistence or exacerbation of negative and depressive symptoms, and vice versa. Such reciprocal effects underscore the importance of longitudinal designs and holistic interventions that address both symptomatic and QoL domains.

Previous meta-analyses have shown that the effects of psychological and pharmacological interventions on functioning and QoL in individuals at CHR-P are generally modest [64, 65]. This limited efficacy may partly reflect the clinical heterogeneity within this population. In particular, it has been suggested that baseline symptom severity – especially negative and depressive symptoms – may moderate treatment response [66]. Although the current meta-analysis was not able to assess this moderating effect due to the lack of stratified data, future studies should explore whether greater symptom severity at baseline predicts differential improvements in QoL following intervention. Clarifying this relationship could help tailor early interventions more effectively to individual clinical profiles.

Another key finding in our study is the improvement in QoL observed at 1-year and 2- to 3-year follow-ups among individuals at CHR-P. However, the magnitude and robustness of these longitudinal effects varied substantially across follow-up intervals and analyses. This improvement could be partially explained by interventions applied in some of the analyzed samples, such as cognitive behavioral therapy alone or combined with pharmacological options like selective serotonin reuptake inhibitors (e.g., fluoxetine), versus monitoring conditions [46, 48]. However, not all the samples included in the meta-analysis belong to interventional studies. In addition to the impact of specific interventions, developmental aspects related to the age of individuals at CHR-P may also play a role in the observed improvement. Adolescence and early adulthood are critical periods for the acquisition and consolidation of social, academic, and occupational skills. Intervening at this stage may allow individuals to maintain or improve functioning and perceived QoL. In contrast to adults with established psychotic disorders, whose outcomes are often complicated by chronicity, relapses, medication side effects, and hospitalizations, individuals at CHR-P are still at a stage of neurodevelopmental plasticity that may result in an improvement of symptoms or recovery [62]. These maturational processes, combined with potential spontaneous symptom remission and psychosocial adjustment, may contribute to the observed short- to medium-term improvements in longitudinal data.

However, longitudinal improvements in QoL may also reflect measurement-related artifacts rather than true clinical change. These include regression to the mean in samples selected for poor baseline functioning, response-shift or repeated-measurement effects, selective attrition of participants with poorer outcomes, and inflation of standardized effect sizes due to heterogeneous instruments and restricted score ranges. Together, these factors further support a guarded interpretation of long-term QoL trajectories in CHR-P populations. Previous research has suggested that the low transition rate in individuals at CHR-P, along with the frequency and intensity of psychotic experiences, could indicate either a natural recovery process or question the validity of the CHR-P concept itself [45, 48]. Some authors argue that the transition to psychosis is a key variable in this context. However, it is equally important to focus on other aspects of psychopathology to ensure that young people with mental health problems receive the appropriate care [49]. In relation to this, previous research [1] found that positive and negative symptoms, depressive symptoms, and functioning consistently improved within the first 2 years. However, in another work by the same author [67], it was found that individuals at CHR-P who did not transition to psychosis improved in various outcomes, but more than half (48.7%) did not achieve remission. In fact, those individuals at CHR-P who do not transition to a FEP remain significantly impaired in their general function [68]. Therefore, it is necessary to investigate not only conversion to psychosis but also QoL and functionality as prognostic factors in individuals at CHR-*P.*

### Clinical implications

The findings of this meta-analysis have important clinical implications for interventions in individuals at CHR-P. Our results indicate that poor QoL in this population is closely related to depressive and negative symptoms, as well as functional impairments. Consequently, treatments targeting these symptoms may improve QoL and serve as crucial components of early intervention strategies, as they represent a core vulnerability for these individuals [69]. Moreover, it is imperative to assess treatable comorbidities, such as depression and anxiety, which may further compromise QoL, and to provide appropriate interventions for these conditions [70]. Beyond affective symptoms, other comorbid conditions, such as substance use and cognitive impairments, may also impact QoL in CHR-P individuals. However, these factors were not consistently reported in the included studies. Future research should explore how a broader range of clinical and neurocognitive variables may influence functioning and subjective well-being in this population. Although current clinical guidelines recommend CBT as a preventive intervention for individuals at CHR-P [71], evidence remains inconclusive regarding the superiority of specific treatments over others [72]. Therefore, interventions that prioritize functional recovery and QoL improvements should remain a focus, as supported by our findings and previous research [73, 74]. Promising alternatives include family-based therapies and structured support in educational and vocational settings, such as individual placement and support models [75]. Mindfulness-based interventions have shown preliminary effectiveness in reducing symptom-related distress and improving social functioning and well-being [76, 77]. Physical exercise interventions are also feasible and potentially beneficial across a range of outcomes, including cognition, depressive symptoms, and functional capacity [78]. In addition, early pharmacological strategies such as cannabidiol (CBD) have demonstrated initial promise in modulating psychotic symptoms and affective distress [79].

### Limitations

This study has several limitations. First, while a considerable number of studies were included in the meta-analysis, data availability for longer follow-up periods was limited, with only a few studies reporting outcomes beyond 24 months. Second, although QoL is a key outcome in clinical practice for neuropsychiatric disorders [80], it is often a secondary focus in research, leading to variability in measurement approaches. Additionally, QoL is a multidimensional construct that is assessed with multiple scales depending on whether it pertains to objective, subjective, or health-related QoL [56], which complicates the understanding of the results of this variable. This heterogeneity made it difficult to extract and analyze data on particular aspects of QoL in a consistent manner. As a result, our findings primarily reflect global QoL scores, rather than specific dimensions such as physical health, social relationships, or psychological well-being. Future studies should prioritize reporting and analyzing domain-specific QoL outcomes using harmonized instruments and approaches. In addition, although no non-English studies were retrieved by the search strategy, the restriction to English-language publications may still introduce a potential language bias, as relevant studies published in other languages may not be indexed in the searched databases. Third, despite applying meta-regression analyses to address heterogeneity, variability across studies remained a factor that could influence the robustness of the findings, with I^2^ values exceeding 90% across several analyses, indicating substantial between-study variability. In particular, some individuals at CHR-P received preventive interventions (e.g., cognitive behavioral therapy, pharmacological treatments) or were recruited through early intervention services, which may have positively influenced their QoL outcomes and introduced variability across studies. Additionally, heterogeneity in QoL constructs and measurement instruments, study design (cross-sectional, longitudinal, and interventional), and follow-up duration likely further contributed to the observed variability. Fourth, the data for certain meta-regression analyses – particularly those examining depressive symptoms, negative symptoms, and positive symptoms – were limited, which constrained our ability to explore their influence in more depth. Moreover, the absence of statistically significant meta-regression findings should be interpreted cautiously, as study-level meta-regression is known to be underpowered in the presence of few studies and restricted covariate ranges. Fifth, although some of the included studies reported whether CHR individuals transitioned to psychosis or not, they did not provide stratified data on QoL based on transition status. This prevented us from conducting a subgroup meta-analysis comparing baseline QoL between those who transitioned and those who did not. Finally, several methodological considerations related to the meta-analytic approach warrant mention. Random-effects models were estimated using the DerSimonian and Laird method, as implemented in CMA. Although Hartung–Knapp/Sidik–Jonkman adjustments are often recommended for random-effects meta-analyses with small numbers of studies and high heterogeneity, this option is not implemented in the software used and can therefore not be applied. As a result, confidence intervals in some analyses may be relatively liberal. Nevertheless, sensitivity analyses using alternative variance estimation (REML) and complementary assessments of publication bias yielded materially similar results, supporting the robustness of the main findings. Despite this, the very high heterogeneity observed across several analyses underscores the need for cautious interpretation of pooled estimates. In addition, assessment of publication bias was limited, as Egger’s test is underpowered in the presence of few studies and substantial heterogeneity, and therefore cannot reliably exclude small-study effects.

## Conclusions

This meta-analysis demonstrates that individuals at CHR-P exhibit worse QoL compared to HC, with clinical symptoms playing a significant role in this deterioration. Longitudinal data suggest possible improvements in QoL over time; however, evidence at longer follow-up intervals is sparse, heterogeneous, and sensitive to influential single studies. These findings underscore the importance of prioritizing QoL and functional recovery in interventions for individuals at CHR-P. Overall, conclusions regarding long-term QoL trajectories should be drawn cautiously, and more longitudinal studies are needed to clarify other factors that may affect QoL and interventions aimed at improving this aspect, given its clinical impact and the importance it holds in the lives of these individuals.

## Supporting information

10.1192/j.eurpsy.2026.10170.sm001 Camacho et al. supplementary material Camacho et al. supplementary material

## Data Availability

The data that support the findings of this study are available from the corresponding author upon reasonable request.

## Supplementary material

The supplementary material for this article can be found at http://doi.org/10.1192/j.eurpsy.2026.10170.
