# Supplementary material for: Quality of life in individuals at clinical high risk for psychosis: A systematic review and meta-analysis
Source: Eur Psychiatry. 2026 Feb 18;69(1):e30. doi: 10.1192/j.eurpsy.2026.10170 (PMC13122532; doi:10.1192/j.eurpsy.2026.10170)
Supplement: Camacho et al. supplementary material [file S0924933826101709sup001.docx]

**SUPPLEMENTARY MATERIAL**

**eTable 1:** PRISMA 2020 statement and checklist page 2-4

**eTable 2:** MOOSE checklist page 5-7

**Supplementary Table S1**: Overlapping cohorts and handling of duplicate publications page 8

**eTable 3**: Definitions and instruments employed to define outcomes page 9

**eTable 4:** Risk of bias (quality) assessment using the modified Newcastle Ottawa Scale for cohort and cross-sectional studies page 10

**eTable 5:** Quality assessment: NOS results page 11-12

**eTable 6:** Publication bias: Egger’s test results pages 12

**eTable 7:** Subgroup analyses – CHR-P VS HC QoL page 13

**eTable 8:** Subgroup analyses – CHR-P longitudinal QoL page 13

**eTable 9:** Meta-regressions page 14

**eTable 10**: QoL instruments, domains reported, and feasibility of domain-level quantitative synthesis pages 14-17

**eTable 11**: Longitudinal meta-analyses stratified by QoL instrument family pages 17-18

**eMethods 1:** CHR assessment tools page 19

**eMethods 2:** QoL assessment tools page 19

**eFigure 1:** Funnel plot – QoL in CHR-P vs HC page 20

**eFigure 2:** Funnel plot – QoL longitudinal progression at one year page 21

**eFigure 3**: Funnel plot – QoL longitudinal progression at two years page 22

**eFigure 4**: Leave-one-out sensitivity analysis for baseline comparisons page 23

**eFigure 5:** Leave-one-out sensitivity analysis for 1-year follow-up page 24

**eFigure 6**: Leave-one-out sensitivity analysis for 2-year follow-up page 25

**References** page 26

**This supplementary material has been provided by the authors to give readers additional information about their work.**

**eTable 1: Prisma 2020 Statement and Checklist (**[**Page et al., 2021**](#_ENREF_13)**)**

| **Section and Topic** | **Item #** | **Checklist item** | **Location where item is reported** |
| --- | --- | --- | --- |
| **TITLE** | | |  |
| Title | 1 | Identify the report as a systematic review. | Page 1 |
| **ABSTRACT** | | |  |
| Abstract | 2 | See the PRISMA 2020 for Abstracts checklist. | Page 2 |
| **INTRODUCTION** | | |  |
| Rationale | 3 | Describe the rationale for the review in the context of existing knowledge. | Page 3 |
| Objectives | 4 | Provide an explicit statement of the objective(s) or question(s) the review addresses. | Page 3 |
| **METHODS** | | |  |
| Eligibility criteria | 5 | Specify the inclusion and exclusion criteria for the review and how studies were grouped for the syntheses. | Page 5 |
| Information sources | 6 | Specify all databases, registers, websites, organisations, reference lists and other sources searched or consulted to identify studies. Specify the date when each source was last searched or consulted. | Page 5 |
| Search strategy | 7 | Present the full search strategies for all databases, registers and websites, including any filters and limits used. | Page 5 |
| Selection process | 8 | Specify the methods used to decide whether a study met the inclusion criteria of the review, including how many reviewers screened each record and each report retrieved, whether they worked independently, and if applicable, details of automation tools used in the process. | Page 5-6 |
| Data collection process | 9 | Specify the methods used to collect data from reports, including how many reviewers collected data from each report, whether they worked independently, any processes for obtaining or confirming data from study investigators, and if applicable, details of automation tools used in the process. | Page 6 |
| Data items | 10a | List and define all outcomes for which data were sought. Specify whether all results that were compatible with each outcome domain in each study were sought (e.g. for all measures, time points, analyses), and if not, the methods used to decide which results to collect. | Page 6 |
|  | 10b | List and define all other variables for which data were sought (e.g. participant and intervention characteristics, funding sources). Describe any assumptions made about any missing or unclear information. | Page 6 |
| Study risk of bias assessment | 11 | Specify the methods used to assess risk of bias in the included studies, including details of the tool(s) used, how many reviewers assessed each study and whether they worked independently, and if applicable, details of automation tools used in the process. | Page 6, eTable 3 & 4 |
| Effect measures | 12 | Specify for each outcome the effect measure(s) (e.g. risk ratio, mean difference) used in the synthesis or presentation of results. | Page 7 |
| Synthesis methods | 13a | Describe the processes used to decide which studies were eligible for each synthesis (e.g. tabulating the study intervention characteristics and comparing against the planned groups for each synthesis (item #5)). | Page 7 |
|  | 13b | Describe any methods required to prepare the data for presentation or synthesis, such as handling of missing summary statistics, or data conversions. | Page 7 |
|  | 13c | Describe any methods used to tabulate or visually display results of individual studies and syntheses. | Page 7 |
|  | 13d | Describe any methods used to synthesize results and provide a rationale for the choice(s). If meta-analysis was performed, describe the model(s), method(s) to identify the presence and extent of statistical heterogeneity, and software package(s) used. | Page 7 |
|  | 13e | Describe any methods used to explore possible causes of heterogeneity among study results (e.g. subgroup analysis, meta-regression). | Page 7 |
|  | 13f | Describe any sensitivity analyses conducted to assess robustness of the synthesized results. | Page 7 |
| Reporting bias assessment | 14 | Describe any methods used to assess risk of bias due to missing results in a synthesis (arising from reporting biases). | Page 7 |
| Certainty assessment | 15 | Describe any methods used to assess certainty (or confidence) in the body of evidence for an outcome. | Page 7 |
| **RESULTS** | | |  |
| Study selection | 16a | Describe the results of the search and selection process, from the number of records identified in the search to the number of studies included in the review, ideally using a flow diagram. | Page 8, Figure 1 |
|  | 16b | Cite studies that might appear to meet the inclusion criteria, but which were excluded, and explain why they were excluded. | NA |
| Study characteristics | 17 | Cite each included study and present its characteristics. | Table 1 |
| Risk of bias in studies | 18 | Present assessments of risk of bias for each included study. | NA |
| Results of individual studies | 19 | For all outcomes, present, for each study: (a) summary statistics for each group (where appropriate) and (b) an effect estimate and its precision (e.g. confidence/credible interval), ideally using structured tables or plots. | Table 1, Figure 2 & 3 |
| Results of syntheses | 20a | For each synthesis, briefly summarise the characteristics and risk of bias among contributing studies. | Page 8 & 9, Table 1 |
|  | 20b | Present results of all statistical syntheses conducted. If meta-analysis was done, present for each the summary estimate and its precision (e.g. confidence/credible interval) and measures of statistical heterogeneity. If comparing groups, describe the direction of the effect. | Page 11, Figures 2 & 3 |
|  | 20c | Present results of all investigations of possible causes of heterogeneity among study results. | Page 11, eTable 5 |
|  | 20d | Present results of all sensitivity analyses conducted to assess the robustness of the synthesized results. | Page 11 |
| Reporting biases | 21 | Present assessments of risk of bias due to missing results (arising from reporting biases) for each synthesis assessed. | Page 8, eTable 3 & 4 |
| Certainty of evidence | 22 | Present assessments of certainty (or confidence) in the body of evidence for each outcome assessed. | Page 11 |
| **DISCUSSION** | | |  |
| Discussion | 23a | Provide a general interpretation of the results in the context of other evidence. | Pages 12-13 |
|  | 23b | Discuss any limitations of the evidence included in the review. | Page 14 |
|  | 23c | Discuss any limitations of the review processes used. | Page 14 |
|  | 23d | Discuss implications of the results for practice, policy, and future research. | Page 12-13 |
| **OTHER INFORMATION** | | |  |
| Registration and protocol | 24a | Provide registration information for the review, including register name and registration number, or state that the review was not registered. | Page 5 |
|  | 24b | Indicate where the review protocol can be accessed, or state that a protocol was not prepared. | Page 5 |
|  | 24c | Describe and explain any amendments to information provided at registration or in the protocol. | N/A |
| Support | 25 | Describe sources of financial or non-financial support for the review, and the role of the funders or sponsors in the review. | Page 14 |
| Competing interests | 26 | Declare any competing interests of review authors. | Page 15 |
| Availability of data, code and other materials | 27 | Report which of the following are publicly available and where they can be found: template data collection forms; data extracted from included studies; data used for all analyses; analytic code; any other materials used in the review. | Page 16 |

**eTable 2: MOOSE** checklist ([Brooke et al., 2021](#_ENREF_5)).

| Criteria | | Brief description of how the criteria were handled in the meta-analysis | |
| --- | --- | --- | --- |
| Reporting of background should include | | | |
| √ | Problem definition | | No meta-analysis has specifically examined how quality of life persists in individuals at CHR-P, including those who transition to psychosis and those who do not and comparing with healthy controls. |
| √ | Hypothesis statement | | The hypothesis was that CHR-P individuals would have lower QoL compared to control subjects, and the persistence of poorer QoL would remain longitudinally. |
| √ | Description of study outcomes | | Study outcomes were focused on reductions and longitudinal changes in QoL scores and were detailed in the results section and supplementary materials. |
| √ | Type of exposure or intervention used | | QoL is assessed through a combination of self-reported measures, clinical evaluations, and objective indicators that together provide a comprehensive picture of an individual's functioning and well-being. |
| √ | Type of study designs used | | Cross-sectional and longitudinal studies were selected. |
| √ | Study population | | Participants were individuals at CHR-P identified through established psychometric instruments. |
| Reporting of search strategy should include | | | |
| √ | Qualifications of searchers | | The credentials of the investigators are indicated in the author list. |
| √ | Search strategy, including time period included in the synthesis and keywords | | We performed a multi-step literature search using keywords described in methods section: until 11^th^ May 2024. |
| √ | Databases and registries searched | | PubMed, PsycINFO, Web of Science, and grey literature sources were searched. |
| √ | Use of hand searching | | References of systematic reviews or meta-analyses that were screened during literature search and the references from the included studies were manually searched |
| √ | List of citations located and those excluded, including justifications | | Details of the literature search process are outlined in the results section and PRISMA flowchart |
| √ | Method of addressing articles published in languages other than English | | Only articles in English language were selected |
| √ | Method of handling abstracts and unpublished studies | | Unpublished studies, conference abstracts, and clinical case reports were excluded |
| √ | Description of any contact with authors | | We did not contact authors |
| Reporting of methods should include | | | |
| √ | Description of relevance or appropriateness of studies assembled for assessing the hypothesis  to be tested | | Detailed inclusion and exclusion criteria are described in the methods section |
| √ | Rationale for the selection and coding of data | | Data extraction was based on relevance to intervention type, study design, and outcome measures. |
| √ | Assessment of confounding | | Confounding factors were assessed through meta-regressions. |
| √ | Assessment of study quality, including blinding of quality assessors; stratification or regression on possible predictors of study results | | The quality of included studies was evaluated using NOS (Newcastle Ottawa Scale) |
| √ | Assessment of heterogeneity | | Heterogeneity was assessed with the I^2^ index |
| √ | Description of statistical methods in sufficient detail to be replicated | | A random-effects meta-analysis was used. Heterogeneity among study point estimates was assessed using Q statistics. The proportion of the total variability in the effect size estimates was evaluated with the I^2^ index |
| √ | Provision of appropriate tables and graphics | | We included the PRISMA flow-chart to describe the literature search and its results |
| Reporting of results should include | | | |
| √ | Table summarizing individual study estimates and overall estimate | | We reported this in the results |
| √ | Table giving descriptive information for each study included | | We have presented descriptive information for each study in the tables and as supplementary material |
| √ | Results of sensitivity testing | | We have reported subgroup analyses in the supplementary material |
| √ | Indication of statistical uncertainty of findings | | Confidence intervals and p-values are reported for all effect sizes. |
| Reporting of discussion should include | | | |
| √ | Quantitative assessment of bias | | Publication bias was assessed using visual inspection of funnel plots and Egger’s test. |
| √ | Justification for exclusion | | We excluded studies based on the rationale of the meta-analysis |
| √ | Assessment of quality of included studies | | The quality of the studies was assessed and reported |
| Reporting of conclusions should include | | | |
| √ | Consideration of alternative explanations for observed results | | We have addressed this point in the discussion section |
| √ | Generalization of the conclusions | | We have addressed this point in the discussion section |
| √ | Guidelines for future research | | We have addressed this point in the discussion section |
| √ | Disclosure of funding source | | The funding source is disclosed in the acknowledgements section |

**Supplementary Table S1. Identification of overlapping cohorts and handling of duplicate publications**

| Cohort (program/city) | Publications identified | Contribution to meta-analyses | How overlap was handled/ Rationale |
| --- | --- | --- | --- |
| PRODS cohort (Finland) | Grano et al., 2009; Grano et al., 2011; Grano et al., 2014 | Grano et al., 2014 | Same cohort with multiple reports; the largest and most comprehensive publication was selected. Earlier reports with smaller samples were not entered in the same MA. |
| Copenhagen Hihg Riks Cohort (Denmark) | Glenthoj et al., 2020a; Glenthoj et al., 2020b | Glenthoj et al., 2020a | Overlapping sample; the longitudinal report with larger N was included. The cross-sectional report was not entered to avoid overlap within the same MA. |
| Seoul CHR Cohort (South Korea) | Kim et al., 2013; Kim et al., 2019 | Not included in MA | Both publications derived from the same cohort. Neither contributed data to any quantitative meta-analysis, thus no double-counting occurred. |
|  |  |  |  |

**eTable 3**: **Definitions and instruments employed to define outcomes**

| **Outcome** | **Definition/ Instruments Used** |
| --- | --- |
| Positive symptoms | Positive and Negative Syndrome Scale (PANSS) ([Kay et al., 1987](#_ENREF_8))  Scale for the Assessment of Positive Symptoms (SAPS) ([Andreasen, 1984](#_ENREF_2))  Brief Psychiatric Rating Scale (BPRS), positive component ([Overall & Gorham, 1988](#_ENREF_12))  Structured Interview for Psychosis-Risk Syndromes (SIPS) ([Miller (1999)](#_ENREF_9) |
| Negative symptoms | Positive and Negative Syndrome Scale (PANSS) ([Kay et al., 1987](#_ENREF_8))  Scale for the Assessment of Negative Symptoms (SANS) ([Andreasen, 1983](#_ENREF_1))  Structured Interview for Psychosis-Risk Syndromes (SIPS) ([Miller (1999)](#_ENREF_9)  Comprehensive Assessment of At Risk mental state (CAARMS) ([Yung (2005)](#_ENREF_16) |
| Depressive symptoms | Montgomery–Åsberg Depression Rating Scale (MADRS) ([Montgomery & Asberg, 1979](#_ENREF_10))  Hamilton Rating Scale for Depression (HDRS) ([HAMILTON, 1960](#_ENREF_7))  Beck Depression Inventory (BDI) ([Beck et al., 1996](#_ENREF_3))  Positive and Negative Syndrome Scale (PANSS) ([Kay et al., 1987](#_ENREF_8))  Quick Inventory of Depressive Symptoms (QIDS) ([Rush et al. (2003)](#_ENREF_15)  Symptom Checklist-90 (SCL-90) ([Derogatis (1973)](#_ENREF_6) |
| Functioning | Global Assessment of Functioning (GAF) ([Piersma & Boes, 1997](#_ENREF_14))  Social and Occupational Functioning Assessment Scale (SOFAS) ([Morosini et al., 2000](#_ENREF_11))  Social Adaptation Self-evaluation Scale (SASS) ([Bosc (1997)](#_ENREF_4) |

**eTable 4. Risk of bias (quality) assessment using the modified Newcastle Ottawa Scale for cohort and cross-sectional studies.**

| **Domain** | **Criteria** | **Maximum Score** |
| --- | --- | --- |
| Cohort studies | | |
| 1 | Representativeness of exposed cohort (e.g., total population or random sample, selected group) | 1 |
| 2 | Method used to ascertain exposure is robust? | 1 |
| 3 | Exposed and unexposed are matched or there is an adjustment for confounding factors? | 2 |
| 4 | Assessment of outcome was blind to exposure status or used record linkage, were robust tools used? | 2 |
| 5 | Follow-up period was sufficiently long for outcomes to occur? | 1 |
| 6 | Loss to follow-up rate is reported, low (<30%), and same in exposed and non-exposed? | 1 |
| Cross-sectional studies | | |
| 1 | Sample representative of target sample | 2 |
| 2 | Sample size justified and satisfactory | 1 |
| 3 | Non-response rate is defined satisfactory, and characteristics of responders/non-responders compared? | 1 |
| 4 | Ascertainment of exposure is valid and/or well described? | 1 |
| 5 | Assessment of outcome with robust tool and/or record linkage? | 2 |
| 6 | Outcome per group reported appropriately? | 1 |

Domains correspond to NOS items assessing selection (Domains 1-2), comparability/confounding (Domain 3), and outcome-related biases including detection and attrition (Domains 4-6).

**eTable 5: Quality assessment results using Newcastle Ottawa Scale (NOS)**

| **First author and year of publication** | **Selection: representativeness** | **Selection: exposure ascertainment** | **Comparability (confounding control)** | **Outcome assessment (blinding/tools)** | **Follow-up duration** | **Attrition/loss to follow-up** | **TOTAL** |
| --- | --- | --- | --- | --- | --- | --- | --- |
| Addington, J. (2008) | 2 | 1 | 0 | 1 | 2 | 1 | 7 |
| Bechdolf, A. (2005) | 1 | 1 | 0 | 1 | 2 | 1 | 6 |
| Fallah Zadeh, M. (2023) | 1 | 1 | 0 | 1 | 2 | 1 | 6 |
| Francey, S. (2005) | 2 | 1 | 0 | 1 | 2 | 1 | 7 |
| Glenthoj, L.B. (2020a) | 1 | 1 | 2 | 0 | 1 | 0 | 5 |
| Glenthoj, L. B. (2020b) | 1 | 1 | 0 | 1 | 2 | 1 | 6 |
| Grano, N. (2009) | 0 | 1 | 1 | 0 | 1 | 0 | 3 |
| Grano, N. (2010) | 1 | 1 | 0 | 1 | 2 | 1 | 6 |
| Grano, N. (2014) | 1 | 1 | 0 | 1 | 2 | 1 | 6 |
| Hauser, M. (2009) | 0 | 0 | 0 | 1 | 2 | 1 | 4 |
| Heinze, K. (2018) | 1 | 0 | 0 | 1 | 2 | 1 | 5 |
| Hui, C. (2013) | 1 | 1 | 0 | 1 | 2 | 1 | 6 |
| Kim, H. K. (2019) | 2 | 1 | 0 | 1 | 2 | 1 | 7 |
| Kim, K.R. (2013) | 2 | 1 | 0 | 1 | 2 | 1 | 7 |
| Lin, A. (2013) | 0 | 1 | 0 | 1 | 2 | 1 | 5 |
| Mao, Z. (2023) | 1 | 1 | 0 | 1 | 2 | 1 | 6 |
| McFarlane, W. (2015) | 1 | 1 | 2 | 1 | 1 | 1 | 7 |
| McGorry, P. (2013) | 1 | 1 | 2 | 2 | 1 | 0 | 7 |
| McGorry, P. (2023) | 1 | 1 | 2 | 2 | 1 | 1 | 8 |
| Michel, C. (2019) | 1 | 1 | 2 | 0 | 1 | 1 | 6 |
| Morita, K. (2014) | 0 | 1 | 2 | 0 | 1 | 0 | 4 |
| Morrison, A. (2012) | 1 | 1 | 2 | 1 | 1 | 0 | 6 |
| Nitka, F. (2016) | 1 | 0 | 0 | 1 | 1 | 1 | 4 |
| Ohmuro, N. (2017) | 2 | 1 | 0 | 1 | 2 | 1 | 7 |
| Ortega, L. (2018) | 1 | 1 | 0 | 1 | 2 | 1 | 6 |
| Ruhrmann, S. (2008) | 1 | 1 | 0 | 1 | 2 | 1 | 6 |
| Stain, H. J. (2016) | 1 | 1 | 2 | 1 | 1 | 0 | 6 |
| Svirskis, T. (2007) | 1 | 1 | 0 | 1 | 2 | 1 | 6 |
| Takahashi, T. (2017) | 1 | 0 | 0 | 1 | 2 | 1 | 5 |
| Usui, K. (2022) | 0 | 1 | 2 | 0 | 1 | 0 | 4 |
| Velthorst, E. (2013) | 1 | 1 | 2 | 0 | 1 | 0 | 5 |

**eTable 6: Publication bias: Egger’s test results**

|  | **Intercept** | **SE** | **95% CI** | | **T value** | **P-value** |
| --- | --- | --- | --- | --- | --- | --- |
| **CHR-P vs control** | 7.230 | 3.748 | -1.248 | 15.708 | 1.929 | 0.085 |
| **CHR-P longitudinal (1 year follow-up)** | 6.723 | 4.734 | -4.862 | 18.307 | 1.420 | 0.205 |
| **CHR-P longitudinal (2 years follow-up)** | 9.254 | 7.551 | -23.23 | 41.745 | 1.225 | 0.345 |

**eTable 7: Subgroup analyses- CHR-P vs HC QoL**

| **Group**, subgroup | **No. of**  **Studies** | **N**  **CHR** | **N CTRL** | **Hedges’ g** | | | **z Score** | **P** | **Test for Heterogeneity** | | | **Within subgroup heterogeneity** | |
| --- | --- | --- | --- | --- | --- | --- | --- | --- | --- | --- | --- | --- | --- |
|  |  |  |  | **Mean** | **95 CI** | |  |  | **Q** | **I^2^** | **P** | **Q** | **P** |
| **Continent** | **11** | **789** | **652** | **1.390** | **0.969** | **1.829** | **6.371** | **<0.001** | **126.417** | **92.090** | **<0.001** | 0.221 | 0.638 |
| Europe | 7 | 507 | 417 | 1.325 | 0.911 | 1.740 | 6.269 | <0.001 | 42.817 | 85.987 | <0.001 |  |  |
| Other | 4 | 282 | 235 | 1.600 | 0.534 | 2.665 | 2.942 | 0.003 | 81.260 | 96.308 | <0.001 |  |  |
| **Scale** | **11** | **789** | **652** | **1.390** | **0.969** | **1.829** | **6.371** | **<0.001** | **126.417** | **92.090** | **<0.001** | 1.916 | 0.166 |
| Other | 8 | 631 | 500 | 1.127 | 0.737 | 1.518 | 5.660 | <0.001 | 58.435 | 88.021 | <0.001 |  |  |
| QlS | 3 | 158 | 152 | 2.258 | 0.705 | 3.812 | 2.849 | 0.004 | 53.052 | 96.230 | <0.001 |  |  |
| **Instrument** | **11** | **789** | **652** | **1.390** | **0.969** | **1.829** | **6.371** | **<0.001** | **126.417** | **92.090** | **<0.001** | 1.795 | 0.408 |
| Other | 3 | 326 | 268 | 1.053 | 0.566 | 1.539 | 4.239 | <0.001 | 13.006 | 84.623 | 0.001 |  |  |
| CAARMS | 4 | 252 | 201 | 2.023 | 0.687 | 3.358 | 2.969 | <0.001 | 96.057 | 96.877 | <0.001 |  |  |
| SIPS | 4 | 211 | 183 | 1.144 | 0.700 | 1.587 | 5.055 | <0.001 | 11.906 | 74.803 | 0.008 |  |  |

**eTable 8: Subgroup analyses- CHR-P longitudinal QoL**

| **Group**, subgroup | **No. of**  **Studies** | **N**  **CHR** | **N CHR 1y** | **N CTRL** | **Hedges’ g** | | | **z Score** | **P** | **Test for Heterogeneity** | | | **Within subgroup heterogeneity** | |
| --- | --- | --- | --- | --- | --- | --- | --- | --- | --- | --- | --- | --- | --- | --- |
|  |  |  |  |  | **Mean** | **95 CI** | |  |  | **Q** | **I^2^** | **P** | **Q** | **P** |
| **Continent** | **8** | **780** | **534** | **n/a** | **1.400** | **0.735** | **2.065** | **4.124** | **<0.001** | **306.841** | **97.719** | **<0.001** | 4.096 | 0.043 |
| Europe | 4 | 383 | 267 |  | 0.594 | 0.485 | 0.703 | 10.708 | <0.001 | 1.87 | 0.000 | 0.600 |  |  |
| Other | 4 | 397 | 267 |  | 2.215 | 0.649 | 3.782 | 2.772 | 0.006 | 213.110 | 98.592 | <0.001 |  |  |
| **Scale** | **8** | **780** | **534** |  | **1.400** | **0.735** | **2.065** | **4.124** | **<0.001** | **306.841** | **97.719** | **<0.001** | 0.018 | 0.893 |
| Other | 6 | 518 | 360 |  | 1.489 | -0.311 | 3.289 | 1.621 | 0.105 | 85.611 | 98.832 | <0.001 |  |  |
| QlS | 2 | 262 | 174 |  | 1.356 | 0.650 | 2.062 | 3.765 | <0.001 | 166.864 | 97.004 | <0.001 |  |  |
| **Instrument** | **8** | **780** | **534** |  | **1.400** | **0.735** | **2.065** | **4.124** | **<0.001** | **306.841** | **97.719** | **<0.001** | 2.479 | 0.290 |
| Other | 1 | 28 | 28 |  | 1.659 | 0.785 | 2.533 | 3.721 | <0.001 | 166.216 | 98.195 | <0.001 |  |  |
| CAARMS | 5 | 525 | 337 |  | 1.192 | -0.194 | 2.578 | 1.685 | 0.092 | 66.586 | 96.996 | <0.001 |  |  |
| SIPS | 2 | 227 | 169 |  | 0.882 | 0.445 | 1.318 | 3.959 | <0.001 | 0.000 | 0.000 | 1.000 |  |  |

**eTable 9: Meta-regressions**

1. **Meta-Regression CHR-P vs control**

|  | **No. of**  **Studies (REV)** | **β Coefficient** | **SE** | **95%CI** | | **Z-Value** | **P value** |
| --- | --- | --- | --- | --- | --- | --- | --- |
| **Age** | 11 | -0.003 | 0.071 | -0.142 | 0.137 | -0.04 | 0.969 |
| **% females** | 11 | -0.011 | 0.017 | -0.044 | 0.022 | -0.66 | 0.512 |
| **Functioning** | 6 | 0.088 | 0.157 | -0.221 | 0.396 | 0.56 | 0.578 |
| **Positive symptoms** | 6 | -0.035 | 0.241 | -0.508 | 0.437 | -0.15 | 0.884 |
| **Negative symptoms** | 6 | 0.536 | 0.317 | -0.086 | 1.158 | 1.69 | 0.091 |
| **Quality assessment** | 11 | 0.654 | 0.509 | -0.345 | 1.653 | 1.28 | 0.199 |

1. **Meta-Regression CHR longitudinal outcomes (1 year).**

|  | **No. of**  **Studies** | **β Coefficient** | **SE** | **95%CI** | | **Z-Value** | **P value** |
| --- | --- | --- | --- | --- | --- | --- | --- |
| **Age** | 8 | -0.162 | 0.115 | -0.388 | 0.062 | -1.42 | 0.157 |
| **% females** | 8 | -0.007 | 0.045 | -0.095 | 0.082 | -0.15 | 0.883 |
| **Functioning** | 7 | -0.016 | 0.031 | -0.078 | 0.046 | -0.51 | 0.608 |
| **Quality assessment** | 8 | 0.563 | 0.297 | -0.019 | 1.144 | 1.90 | 0.058 |

**eTable 10: Availability of domain-level QoL data and feasibility of domain-specific synthesis**

| **First author, year** | **QoL assessment tools** | **Domains reported** | **Total score** | **Domain-level SD available** | **Domain-level SD available** |
| --- | --- | --- | --- | --- | --- |
| Addington, 2008 | QLS (role) | Partially. Instrumental role | Yes, instrumental role | **No** | **No (single domain; SD not available)** |
| Bechdolf, 2005 | MSQoL | Partially. Core module | Yes | **Partial** | **No (partial domain reporting; heterogeneous structure)** |
| Fallah Zadeh, 2023 | QLS | Instrumental role, interpersonal relationship, intrapsychic foundation, common objects and activities | Yes | **Yes** | **No** |
| Francey, 2005 | QLS | No | Yes | **No** | **No** |
| Glenthoj, 2020a | AQoL (8D) | No | Yes | **No** | **No** |
| Glenthoj, 2020b | AQoL | No | Yes | **No** | **No** |
| Grano, 2014 | HRQoL 16D | No | Yes | **No** | **No** |
| Grano, 2011 | HRQoL 16D | No | Yes | **No** | **No** |
| Grano, 2009 | HRQoL 16D | No | Yes | **No** | **No** |
| Hauser, 2009 | MSQoL | No. Core module | Yes | **Partial** | **No (SD not consistently available)** |
| Heinze, 2018 | WHOQOL-BREF | No | Yes | **No** | **No** |
| Hui, 2013 | MANSA | Yes. Objective and subjective domains (life in general, health, work and education, finance, leisure, safety, living situation, social and family relations). | Yes | **Yes** | **No (not comparable across studies)** |
| Kim, 2019 | QLS | Yes. Instrumental role, interpersonal relations, intrapsychic foundation, common objects and activities | Yes | **Yes** | **No (not comparable across studies)** |
| Kim, 2013 | QLS | Yes. Instrumental role, interpersonal relations, intrapsychic foundation, common objects and activities | Yes | **Yes** | **No (not comparable across studies)** |
| Lin, 2013 | QLS | No | Yes | **No** | **No (not comparable across studies)** |
| Mao, 2023 | WHOQOL-BREF | Yes. Physical health, psychological health, social relationships, environment | Yes | **Yes** | **No** |
| McFarlane, 2015 | QLS | Partially reported. Instrumental role, intrapsychic foundation, interpersonal relations | Yes | **Yes** | **No** |
| McGorry, 2023 | AQoL | No | Yes | **No** | **No** |
| McGorry, 2013 | QLS | No | Yes | **No** | **No** |
| Michel, 2019 | BMLSS | Yes. Intrinsic, social, external, perspective | Yes | **Yes** | **No** |
| Morita, 2014 | WHOQOL-26 | Yes. Physical health, psychological health, social relationships, environment | Yes | **Yes** | **No** |
| Morrison, 2012 | MANSA | No | Yes | **No** | **No** |
| Nitka, 2016 | HRQoL | Yes. Physical well-being, psychological well-being, autonomy and parents, peers and social support, school environment | Yes | **Yes** | **No (not comparable)** |
| Ohmuro, 2007 | WHOQOL-BREF | Partially reported. Physical health, psychological health, social relationships, environment | Yes | **Yes** |  |
| Ortega, 2018 | EQ-5D | Subjective. Mobility, self-care, daily activities, pain/discomfort, anxiety/depression | Yes | **No** |  |
| Ruhrmann, 2008 | MSQoL | No. Core module (physical health, vitality, psychosocial, affective, material, spare time, general). | Yes | **No** |  |
| Stain, 2016 | QLS | Partially reported. Intrapsychic foundation, interpersonal relations | Yes | **Partial** |  |
| Svirskis, 2007 | QLS | No | Yes | **No** |  |
| Takahasi, 2017 | QLS | No | Yes | **No** |  |
| Usui, 2022 | WHOQOL | Yes. Physical health, psychological health, social relationships, environment | Yes | **Yes** |  |
| Velthorst, 2013 | QLS | No | Yes | **No** |  |

Although several studies reported domain-level QoL outcomes, reporting was highly heterogeneous across instruments, domains, and scoring metrics, and often lacked extractable standard deviations or provided subgroup-specific estimates only. Consequently, domain-level quantitative synthesis or sensitivity analyses were not methodologically feasible.

**eTable11: Longitudinal meta-analyses stratified by QoL instrument family**

| **Instrument family** | **K** | **Effect size** | **interpretation** |
| --- | --- | --- | --- |
| QLS (1y FU) | 2 |  | Feasible |
| AQoL (1y FU) | 2 |  | Feasible |
| WHOQOL (1y FU) | 1 |  | Descriptive |
| Others (1y FU) | 3 |  | Descriptive |
| QLS (2-3y FU) | 1 |  | Descriptive |
| WHOQOL (2-3y FU) | 2 |  | Feasible |
| Other (2-3y FU) | 1 |  | Descriptive |

**eMethods 1:**

**CHR-P instruments included**

The CHR-P state comprises the Ultra-High-Risk state and/or Basic Symptoms.

- The following CHR instruments were considered to define the CHR state: Comprehensive Assessment of At-Risk Mental States (CAARMS), Structured Interview for Psychosis-risk Syndromes (SIPS), Early Recognition Inventory (ERIraos), and Prodromal Risk of Disorder Screen (PRODS). Furthermore, before the development of these instruments, the CHR-P state was defined through the Positive and Negative Syndrome Scale (PANSS) or Brief Psychiatric Rating Scale (BPRS). In our sample, only one small study used BPRS and none used PANSS. Importantly, this study did not contribute data to any quantitative meta-analysis; therefore, no material influence on meta-analytic estimates is expected.
- The following CHR instruments were considered to define BS: Bonn Scale for the Assessment of Basic Symptoms (BSABS), Basel Screening Instrument for Psychosis (BSIP), and Schizophrenia Proneness Instrument - Adult (SPI-A) and Child and Youth (SPI-CY) version.

**eMethods 2:**

**QoL instruments included**

The QoL data was assessed according to validated measurement instruments:

- Quality of life (QLS) (schizophrenia specific instrument), World Health Organization Quality of Life (WHOQOL), Modular Scale of Quality of Life (MSQoL) (mental health instrument), EuroQoL Five Dimensions (EQ-5D), Health Related Quality of Life (HRQoL), Manchester Short Assessment of Quality of Life (MANSA), Brief Multidimensional Life Satisfaction Scale (BMLSS), Assessment of Quality of Life (AQoL).

**eFigure 1: Funnel plot: QoL in CHR vs control**

**eFigure2: Funnel Plot: QoL longitudinal progression at one year**

**eFigure 3: Funnel Plot: QoL longitudinal progression at two years**

**eFigure 4. Leave-one-out sensitivity analysis for baseline comparisons.**


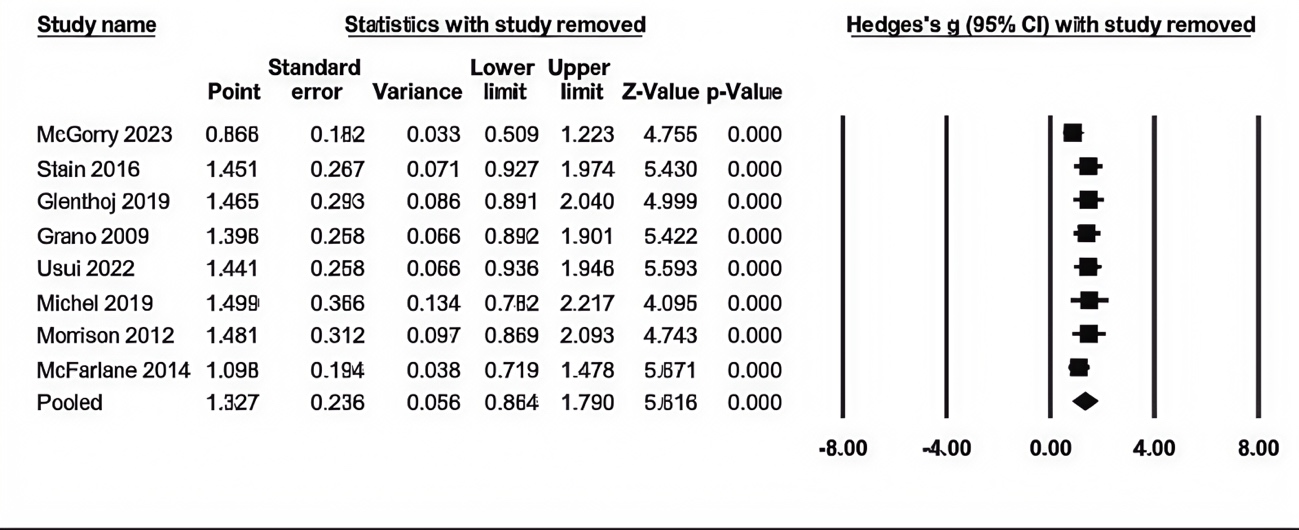


**eFigure 5. Leave-one-out sensitivity analysis for 1-year follow-up.**


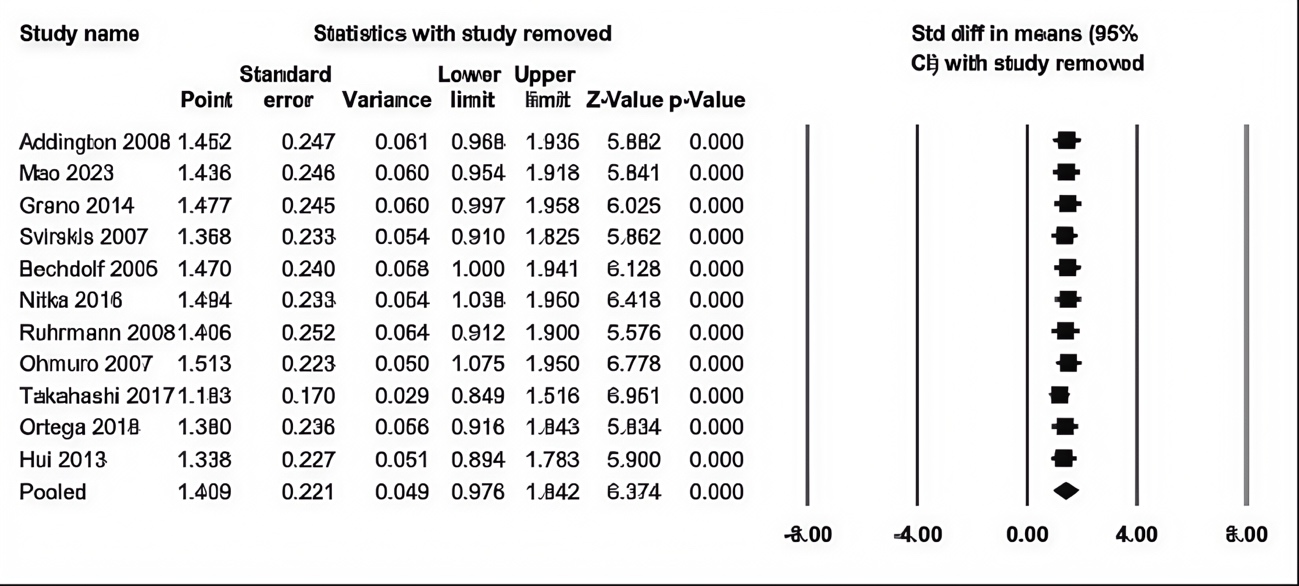


**eFigure 6. Leave-one-out sensitivity analysis for 2-year follow-up.**


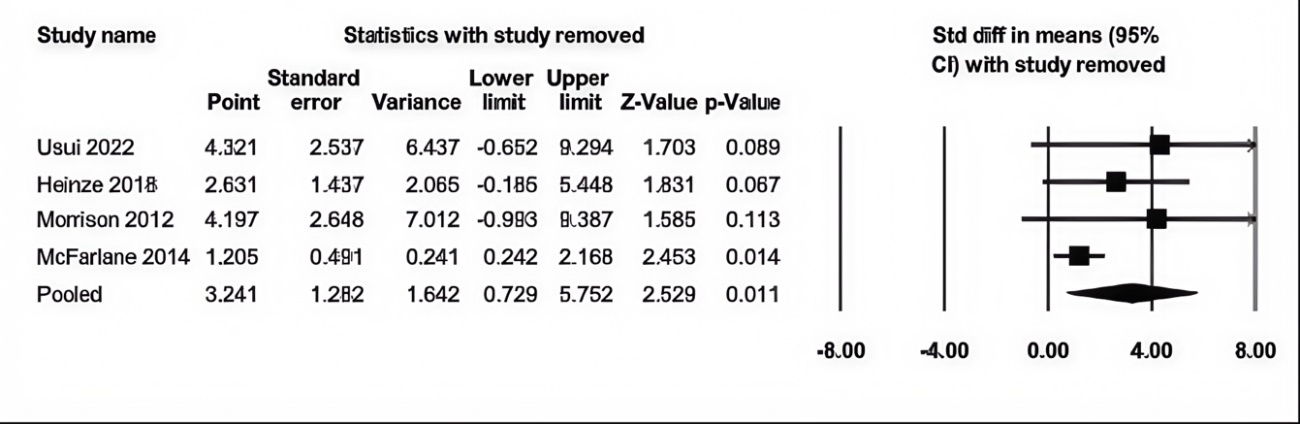


**REFERENCES**

Andreasen, N. (1983). Scale for the Assessment of Negative Symptoms (SANS). In. Iowa City: University of Iowa.

Andreasen, N. (1984). Scale for the Assessment of Positive Symptoms (SAPS). In. Iowa City: University of Iowa.

Beck, A., Steer, R., & Brown, G. (1996). Manual for the beck depression inventory-II. In. Psychological Corporation, San Antonio, TX.

Bosc, M., Dubini, A., & Polin, V. (1997). Development and validation of a social functioning scale, the Social Adaptation Self-evaluation Scale. *European Neuropsychopharmacology*, *7 (1)*, S57-S70.

Brooke, B. S., Schwartz, T. A., & Pawlik, T. M. (2021). MOOSE Reporting Guidelines for Meta-analyses of Observational Studies. *JAMA Surg*, *156*(8), 787-788. <https://doi.org/10.1001/jamasurg.2021.0522>

Derogatis, L. R., Lipman, R. S., & Covi, L. (1973). SCL-90: an outpatient psychiatric rating scale–preliminary report. *Psychopharmacol Bull*, *9 (1)*, 13-28.

HAMILTON, M. (1960). A rating scale for depression. *J Neurol Neurosurg Psychiatry*, *23*, 56-62. <https://doi.org/10.1136/jnnp.23.1.56>

Kay, S. R., Fiszbein, A., & Opler, L. A. (1987). The positive and negative syndrome scale (PANSS) for schizophrenia. *Schizophr Bull*, *13*(2), 261-276. <https://doi.org/10.1093/schbul/13.2.261>

Miller, T. J., McGlashan, T. H., Woods, S. W., Stein, K., Driesen, N., Corcoran, C. M., ... & Davidson, L. (1999). Symptom assessment in schizophrenic prodromal states. *Psychiatric Quarterly*, *70*, 273-287.

Montgomery, S. A., & Asberg, M. (1979). A new depression scale designed to be sensitive to change. *Br J Psychiatry*, *134*, 382-389. <https://doi.org/10.1192/bjp.134.4.382>

Morosini, P. L., Magliano, L., Brambilla, L., Ugolini, S., & Pioli, R. (2000). Development, reliability and acceptability of a new version of the DSM-IV Social and Occupational Functioning Assessment Scale (SOFAS) to assess routine social functioning. *Acta Psychiatr Scand*, *101*(4), 323-329. <https://www.ncbi.nlm.nih.gov/pubmed/10782554>

Overall, J., & Gorham, D. (1988). The Brief Psychiatric Rating Scale (BPRS): recent developments in ascertainment and scaling. *Psychopharmacol Bull*, *24*, 97-99.

Page, M. J., McKenzie, J. E., Bossuyt, P. M., Boutron, I., Hoffmann, T. C., Mulrow, C. D., Shamseer, L., Tetzlaff, J. M., Akl, E. A., Brennan, S. E., Chou, R., Glanville, J., Grimshaw, J. M., Hrobjartsson, A., Lalu, M. M., Li, T., Loder, E. W., Mayo-Wilson, E., McDonald, S.,…Moher, D. (2021). The PRISMA 2020 statement: an updated guideline for reporting systematic reviews. *Bmj*, *372*, n71. <https://doi.org/10.1136/bmj.n71>

Piersma, H. L., & Boes, J. L. (1997). The GAF and psychiatric outcome: a descriptive report. *Community Ment Health J*, *33*(1), 35-41. <https://doi.org/10.1023/a:1022413110345>

Rush, A. J., Trivedi, M. H., Ibrahim, H. M., Carmody, T. J., Arnow, B., Klein, D. N., Markowitz, J. C., Ninan, P. T., Kornstein, S., Manber, R., Thase, M. E., Kocsis, J. H., & Keller, M. B. (2003). The 16-Item Quick Inventory of Depressive Symptomatology (QIDS), clinician rating (QIDS-C), and self-report (QIDS-SR): a psychometric evaluation in patients with chronic major depression. *Biol Psychiatry*, *54*(5), 573-583. <https://doi.org/10.1016/s0006-3223(02)01866-8>

Yung, A. R., Yung, A. R., Pan Yuen, H., Mcgorry, P. D., Phillips, L. J., Kelly, D., ... & Buckby, J. (2005). Mapping the onset of psychosis: the comprehensive assessment of at-risk mental states. *Australian & New Zealand Journal of Psychiatry*, *39(11-12)*, 964-971.
